# TNFR1-d2 carrying the p.(Thr79Met) pathogenic variant is a potential novel actor of TNFα/TNFR1 signalling regulation in the pathophysiology of TRAPS

**DOI:** 10.1038/s41598-021-83539-9

**Published:** 2021-02-18

**Authors:** Cécile Rittore, Déborah Méchin, Elodie Sanchez, Léa Marinèche, Vuthy Ea, Stephan Soler, Marion Vereecke, Aude Mallavialle, Eric Richard, Isabelle Duroux-Richard, Florence Apparailly, Isabelle Touitou, Sylvie Grandemange

**Affiliations:** 1 INSERM U1183, CHU Saint Eloi, IRMB, Univ Montpellier, 80 Avenue Augustin Fliche, 34295 Montpellier Cedex 5, France; 2 Department of Medical Genetics, Rare Diseases and Personalized Medicine, CHU Montpellier, Montpellier, France; 3 IRCM, INSERM, Univ Montpellier, Montpellier, France; 4 Clinical Department for Osteoarticular Diseases, CHU Montpellier, Univ Montpellier, Montpellier, France

**Keywords:** Functional genomics, NF-kappaB

## Abstract

Binding of tumour necrosis factor α (TNFα) to its receptor (TNFR1) is critical for both survival and death cellular pathways. TNFα/TNFR1 signalling is complex and tightly regulated at different levels to control cell fate decisions. Previously, we identified TNFR1-d2, an exon 2-spliced transcript of *TNFRSF1A* gene encoding TNFR1, whose splicing may be modulated by polymorphisms associated with inflammatory disorders. Here, we investigated the impact of *TNFRSF1A* variants involved in TNFR-associated periodic syndrome (TRAPS) on TNFR1-d2 protein expression and activity. We found that TNFR1-d2 could be translated by using an internal translation initiation codon and a de novo internal ribosome entry site (IRES), which resulted in a putative TNFR1 isoform lacking its N-terminal region. The kinetic of assembly of TNFR1-d2 clusters at the cell surface was reduced as compared with full-length TNFR1. Although co-localized with the full-length TNFR1, TNFR1-d2 neither activated nuclear factor (NF)-κB signalling, nor interfered with TNFR1-induced NF-κB activation. Translation of TNFR1-d2 carrying the severe p.(Thr79Met) pathogenic variant (also known as T50M) was initiated at the mutated codon, resulting in an elongated extracellular domain, increased speed to form preassembled clusters in absence of TNFα, and constitutive NF-κB activation. Overall, TNFR1-d2 might reflect the complexity of the TNFR1 signalling pathways and could be involved in TRAPS pathophysiology of patients carrying the p.(Thr79Met) disease-causing variant.

## Introduction

The tumor necrosis factor α (TNFα) pathway is a complex process critically involved in inflammatory responses, cell proliferation, and differentiation^1,2^. TNFα binds to two TNF receptors, TNFR1 and TNFR2, with a major role in TNFα/TNFR1 signalling^3^.

TNFR1 is a membrane protein with an extracellular region of four cysteine-rich domains (CRDs 1 to 4), a transmembrane region and an intracellular “death domain” (DD) involved in signal transduction^4–6^. Once activated by TNFα binding to its extracellular domain, TNFR1 recruits various proteins to form a multiprotein complex that promotes several pathways involved in cell survival or cell death^7–9^. The different TNFα/TNFR1 signalling pathways are complex, and their tight regulation at several levels influences cell fate decisions. The regulation factors include the duration of TNFα exposure^10^, alternative splicing^11^ and post-translational modifications^12,13^, which ensure a balance between cell survival and death. Despite a broad range of data on the regulation of TNFα/TNFR1 signalling pathways, these are not fully understood.

However, the involvement of TNFα/TNFR1 signalling pathways in the pathogenesis of human diseases is clear^14,15^, and their blockade is effective in chronic inflammatory disorders, especially immune diseases^11,12^. In particular, altered TNFα/TNFR1 signalling associated with increased pro-inflammatory responses is involved in the pathogenesis of TNFR-associated periodic syndrome (TRAPS, OMIM 142680), a dominantly inherited autoinflammatory disease characterized by recurrent attacks of fever, abdominal pain, arthritis, and cutaneous manifestations^16,17^. At least 110 different heterozygous disease-causing variants in *TNFRSF1A* gene encoding TNFR1 are responsible for TRAPS; they are predominantly located in the CRD1 and CRD2 domains^18^. Allelic heterogeneity is associated with phenotypic heterogeneity in TRAPS patients^19^. Indeed several studies report that the pathogenic variants p.(Cys62Tyr), p.(Cys81Phe) and p.(Thr79Met) (also known as T50M) cause a severe phenotype, whereas the frequent p.(Pro75Leu) and p.(Arg121Gln) variants are associated with incomplete penetrance and mild phenotypes^20,21^.

Several hypotheses have been proposed for the mechanisms underlying the TRAPS pathophysiology^16,17,22^. Initially, defective TNFR1 receptor shedding, leading to maintained pro-inflammatory responses, was proposed^18^. However, this defect depended on the nature of the variant^23^ and cell type^24^ tested. Several other defects in the signalling pathways of mutated TNFR1 include increased nuclear factor (NF)-κB or mitogen-activated protein kinase (MAPK) activation controlling a sustained survival pathway; hyper-responsiveness to induction with lipopolysaccharide, TNFα or interleukin 1β (IL-1β) leading to overexpression of pro-inflammatory cytokines; or intracellular retention of TNFR1 mainly in endoplasmic reticulum inducing a cellular stress^16,17,22^. All these defects can be strictly linked and one can be the consequence of another. Thus, the pathogenesis of TRAPS seems more complex than anticipated and can depend on the type of variant and cells studied.

A proposed additional level of TNFR1 regulation involves the existence of two alternative transcripts of *TNFRSF1A* gene. Interestingly, the regulation of exons 2 and 6 skipping in TNFR1-d2 and Δ6-TNFR1, respectively, may be modulated by polymorphisms identified as susceptibility markers of inflammatory disorders^25–28^. We showed that the *TNFRSF1A* promoter exon 1 and intron 4 surrounding three single nucleotide polymorphisms (SNPs; rs4149570, rs767455 and rs1800692) have a functional effect on exon 2 splicing^25^. Gregory et al. demonstrated that a SNP located in intron 6 (rs1800693), identified as a susceptibility marker for multiple sclerosis^27^, is associated with exon 6 skipping in the Δ6-TNFR1 transcript. This novel transcript directs the expression of a soluble form of TNFR1, which is capable of TNFα antagonism and might be responsible for worsening multiple sclerosis upon anti-TNF therapy^28^.

Here, we tried to elucidate a potential link between TNFR1-d2 and TRAPS pathogenesis. We first characterized the TNFR1-d2 protein and showed that TNFR1-d2 cDNA can be translated by a cap-independent mechanism, thereby allowing the expression of a shorter form of TNFR1 that lacks the first two CRD domains. Although TNFR1 and TNFR1-d2 were in close subcellular location and could interact, TNFR1-d2 did not activate the transcription factor NF-κB. Importantly, TNFR1-d2 carrying the p.(Thr79Met) disease-causing variant created an alternative translation from mutated Met79, which led to an elongated extracellular domain, increased speed to form preassembled clusters, and a gain of function in spontaneous NF-κB activation. Altogether, our results suggest a possible involvement of TNFR1-d2 in TRAPS pathogenesis.

## Results

### Identification of the TNFR1-d2 translation initiation codon

**Figure 1:**
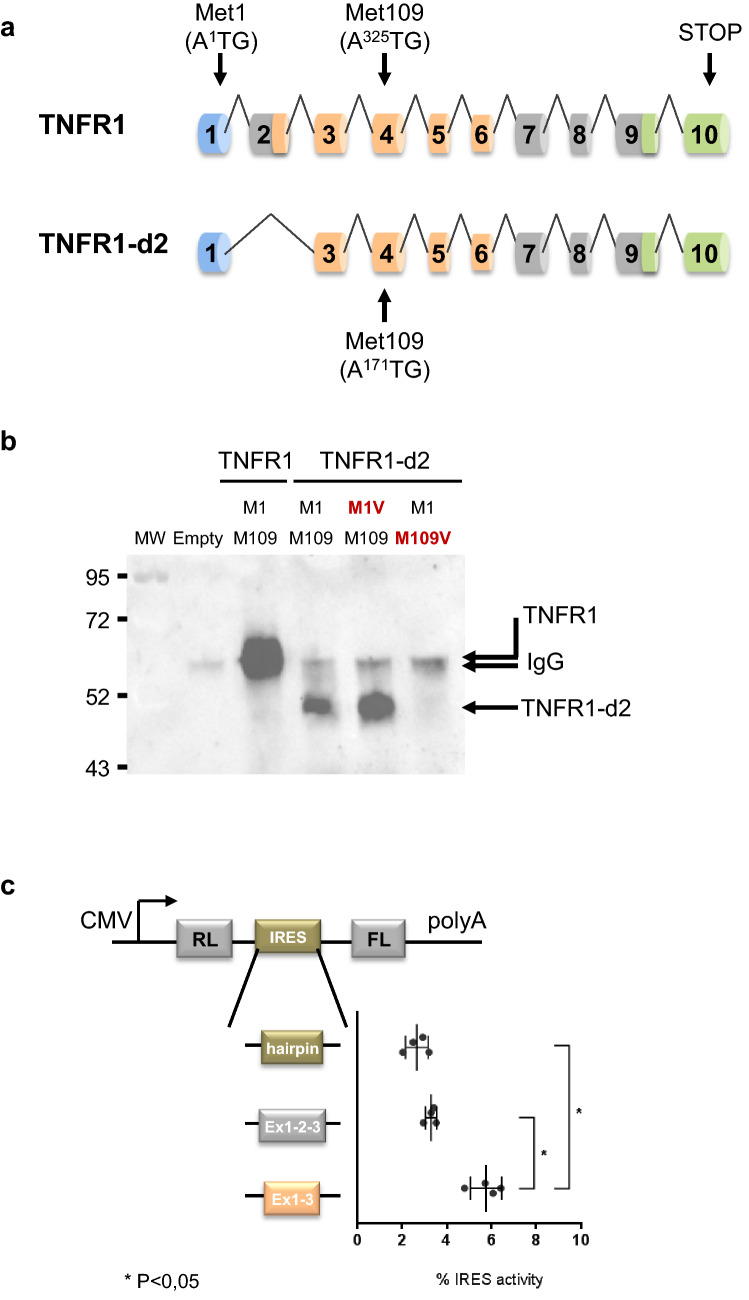
Translation of TNFR1-d2 by an internal initial codon. (**a**) Schematic representation of the TNFR1 and TNFR1-d2 transcripts. Location of the first amino acids (A^1^TG) in exon 1 and the potential initial amino acid for TNFR1-d2 in exon 4 are depicted by arrows (A^325^TG or A^171^TG in TNFR1 or TNFR1-d2 nomenclature, respectively). Exons coding for signal peptide, cysteine-rich domains and death domain are represented in blue, orange, and green cylinders, respectively. (**b**) HEK293T cells were transiently transfected with vectors containing the TNFR1 and TNFR1-d2 cDNAs fused in the C-terminal frame with Flag tag. To identify the initial start codon of TNFR1-d2, the two methionines in exon 1 (c.1A>G p.(Met1Val)) and exon 4 (c.171A>G, p.(Met109Val)) were mutated by directed mutagenesis (in red). Flag-tagged empty vector was transfected as a negative control. After 48 h, cells were collected and proteins were extracted, followed by immunoprecipitation with anti-Flag antibody and immunoblotting with anti-TNFR1 antibody directed against the C-terminal part of TNFR1. MW is the molecular weight marker in kiloDaltons (kDa). (**c**) The top is the schematic representation of the bicistronic vector encoding bicistronic Renilla and Firefly luciferase mRNA (RL and FL, respectively) under control of a CMV promoter. The potential IRES sequence was inserted between the two open reading frames. Below are three vectors used in this experiment: a hairpin sequence in the pCRHL vector^57^ as a negative control, the exon 1 to 3 sequence of TNFR1 and the exon 1 and exon 3 sequences of TNFR1-d2. After co-transfection in HEK293T cells with the β-galactosidase vector, FL values were normalized to RL values, β-galactosidase activity and protein concentration. The ratio of each construct is represented as a percentage of the pCREL construct containing an EMCV IRES^57^ (not represented). Values are mean ± SD from four independent and duplicate transfections and were compared by nonparametric Mann–Whitney U test (GraphPad Prism 6 software). *P < 0.05.

To characterize the TNFR1-d2 protein, we first analysed its expression. Indeed, exon 2 splicing in TNFR1-d2 results in a frameshift and a premature terminal codon in exon 5 when the regular TNFR1 initiation codon that we here call A^1^TG is used (Fig. 1a and Supplementary Fig. S1). Because the corresponding protein would not have any homology with TNFR1 or with other known proteins, we hypothesized that the TNFR1-d2 transcript was translated using the open reading frame 3 by using an alternative internal initiation codon (M109, A^171^TG), located at the end of the CRD2 domain, which restores the C-terminal part of TNFR1 (Supplementary Fig. S1).

To confirm our hypothesis, we cloned the TNFR1-d2 cDNA in the C-terminal frame with the Flag tag and investigated its translation by overexpressing the construct in HEK293T cells. TNFR1-d2 protein migrated lower than TNFR1, at an apparent molecular mass of 49 kDa (Fig. 1b). In agreement with translation by an internal initial codon, no change was observed when we mutated the first methionine to valine (c.1A>G) and no protein expression was detected when Met109 was mutated (c.171A>G in TNFR1-d2, corresponding to c.325A>G in TNFR1). This result suggests that the translation of TNFR1-d2 was initiated at a start codon localized in exon 4, in-frame with TNFR1 and leading to a putative new protein variant of TNFR1 lacking the peptide signal (i.e., the first CRD [CRD1] and the three quarters of CRD2).

### Identification of the putative internal ribosome entry site (IRES) element in TNFR1-d2

An internal initiation of translation can be processed by a cap-independent mechanism, which is based on the presence of a structural sequence on mRNA called the internal ribosome entry site (IRES) downstream of the start codon. Using a bicistronic strategy, we tested the hypothesis of presence of an IRES formed by the junction of exons 1 and 3 only in the transcript TNFR1-d2. TNFR1 (exons 1 to 3) and TNFR1-d2 (exon 1–exon 3) sequences were inserted into the intercistronic region of a luciferase bicistronic vector (Fig. 1c). In these vectors, translation of the upstream Renilla luciferase (RL) occurs via cap-dependent scanning, whereas translation of Firefly luciferase (FL) depends on the presence of an IRES in the intercistronic region. The FL/RL ratio was used to monitor IRES activity. Only the construct containing the TNFR1-d2 sequence significantly increased the second cistron expression as compared with the hairpin sequence as a negative control, and exon 1 to 3 sequence, which suggests the presence of de novo IRES activity formed by the exon 1 and 3 junction in TNFR1-d2.

### Cellular localization of TNFR1 and TNFR1-d2

**Figure 2:**
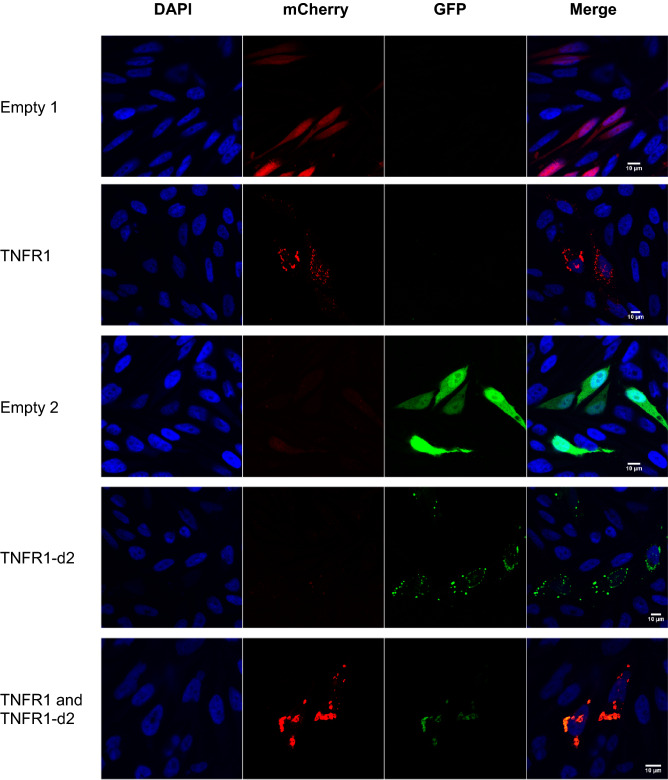
TNFR1-d2 is localized close to TNFR1. Co-localization of TNFR1 and TNFR1-d2 is observed in cells co-transfected with TNFR1-mCherry and TNFR1-d2-GFP vectors by confocal microscopy. HeLa cells were transiently transfected with pcDNA vectors encoding mCherry (empty 1), TNFR1-mCherry, GFP (empty 2), or TNFR1-d2-GFP proteins. Nuclei were counterstained with DAPI (blue) and analysed by confocal microscopy (×63). Representative images of two independent experiments are shown.

**Figure 3:**
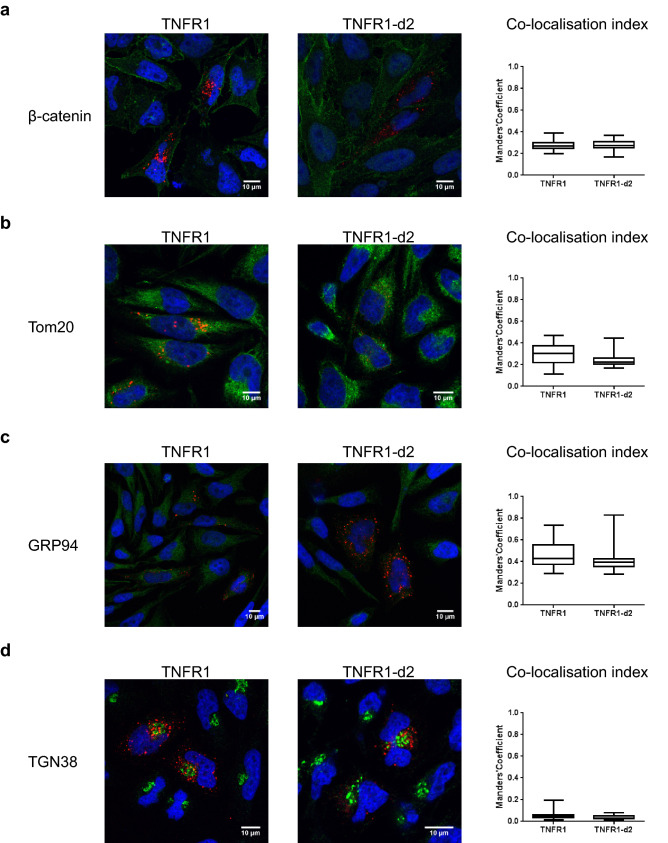
Subcellular location of TNFR1-d2. TNFR1 or TNFR1-d2 fused to C-terminal Flag proteins expressed in HeLa cells. Subcellular co-localization was determined by co-immunofluorescence with a Flag antibody (red) and antibodies (green) for β-catenin (**a**), Tom20 (**b**), GRP94 (**c**) and TGN38 (**d**) proteins to detect membrane junction, mitochondria, endoplasmic reticulum and Golgi apparatus, respectively. After counterstaining with DAPI (blue) cells were visualized by confocal microscopy (×63). Representative zoomed and merged images of two independent experiments are shown (see supplemental Fig. S2 and S3 for non-merged images). On the right, the boxes correspond to the median and minimal and maximum co-localization index of TNFR1 and TNFR1-d2 with the subcellular compartments. At least 25 random fields were analysed and the co-localization index was calculated by Manders’ coefficient representing the fraction of TNFR1 or TNFR1-d2 (red) overlapping the green channel (JaCoP plugin in ImageJ software).

We first determined the cellular localization of TNFR1-d2-GFP with or without transfection with TNFR1-mcherry (Fig. 2). We found no redistribution of TNFR1 or TNFR1-d2 when the proteins were co-expressed as compared with each alone. In addition, we observed a complete cytoplasmic co-localization of TNFR1-d2 and TNFR1 (M1, co-localization index calculated by the Manders’ coefficient^29^ = 0.9, data not shown). Because TNFR1-d2 can be functionally active in a cell compartment different from that of TNFR1 and because different subcellular localizations have been detected in vitro for TNFR1^30^, we compared the subcellular location of TNFR1 and TNFR1-d2. The cDNAs of TNFR1 and TNFR1-d2 fused to the Flag tag were overexpressed in HeLa cells, then underwent immunofluorescence assay with Flag antibody and antibodies for proteins located in specific subcellular compartments. The co-localization index between Flag and membrane or mitochondria staining indicated that TNFR1 and TNFR1-d2 presented occasional co-localization with β-catenin and Tom20 proteins (Fig. 3a,b and Supplementary Fig. S2). TNFR1 and TNFR1-d2 predominantly showed a punctuated cytoplasmic staining, partially close to the glucose-regulated protein 94 (GRP94), which suggests that both proteins were in part co-localized in endoplasmic reticulum (Fig. 3c and Supplementary Fig. S3a). In contrast, we observed no co-localization between TNFR1 or TNFR1-d2 and the Trans-Golgi network protein 38 (TGN38) (Fig. 3d and Supplementary Fig. S3b). Various studies have observed TNFR1 in the Golgi apparatus^31,32^, so we checked this result with another technique. Using a Bodipy tracker, we detected complete co-localization between TNFR1-GFP or TNFR1-d2-GFP and global Golgi apparatus staining (Supplementary Fig. S4). Our results suggested that TNFR1-d2 did not present a specific intracellular location but showed punctuated localization close to TNFR1.

**Figure 4:**
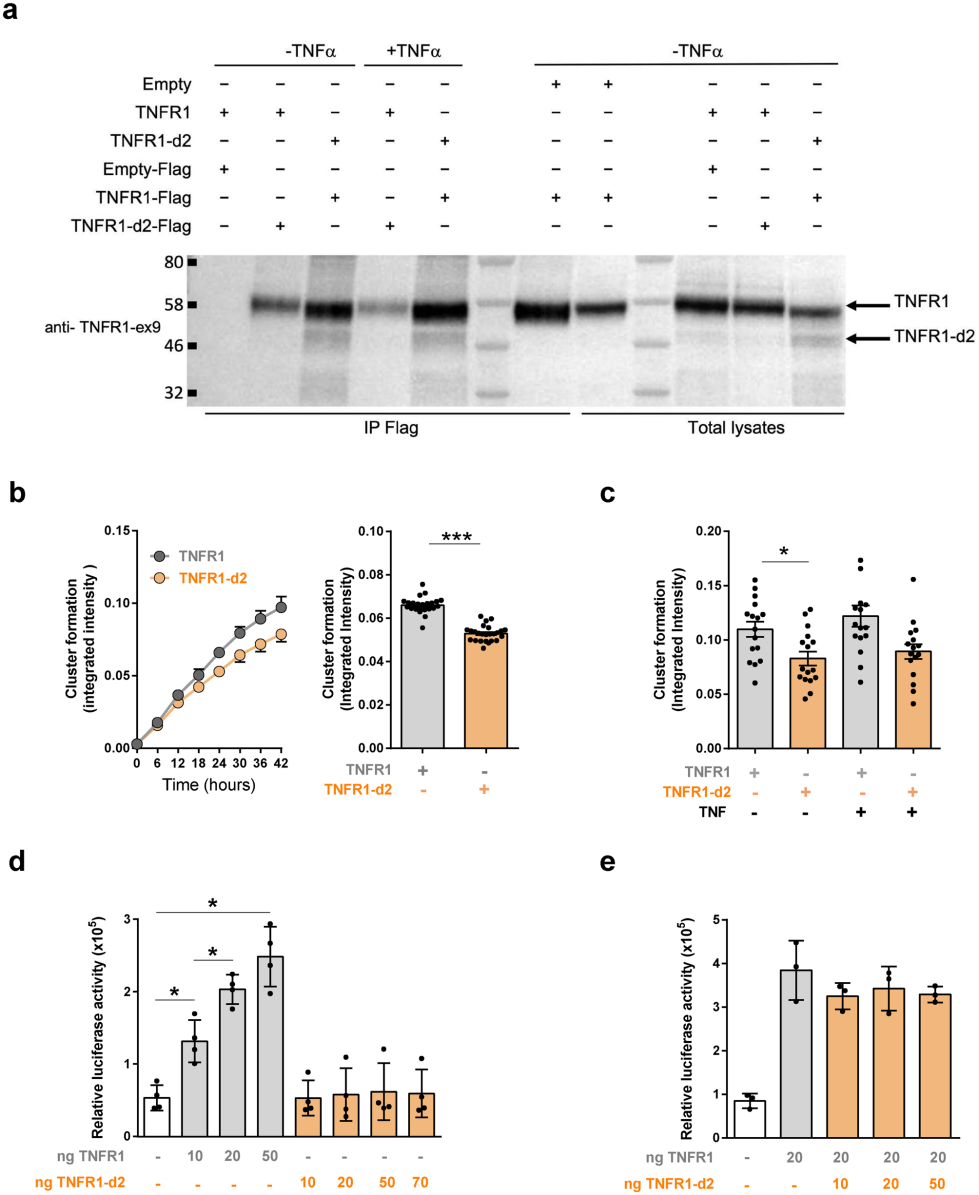
TNFR1-d2 does not affect the NF-κB signalling pathway mediated by TNFR1. (**a**) HEK293T cells were transiently transfected with vectors containing the TNFR1, TNFR1-d2 or empty cDNAs with an alternatively Flag-tagged, in presence and absence of TNFα (10 ng/ml). Immunoprecipitations were performed using anti-flag antibody and the blot was probed with antibody directed against exon9 of TNFR1. Total lysates and co-immunoprecipitations are presented. Protein size markers in kDa are indicated on the left. (**b**,**c**) To determine the dynamics of receptor clusters oligomerization, HEK293T cells were transiently transfected with GFP plasmids of TNFR1 or TNFR1-d2, in presence or absence of TNFα (10 ng/ml). Fluorescent and phase-contrast images were monitored using the IncuCyte live cell imaging system (Essen Bioscience). Histograms represent the GFP intensity of both transfection conditions over 24hours without (**b**) or with TNF stimulation (**c**) in two independent experiments. Each dot corresponds to one well microscopic field. (**d**,**e**) To detect spontaneous activation of the endogenous NF-κB transcription factor, HEK293T cells were transiently transfected with the indicated amount of TNFR1 and TNFR1-d2 vectors either alone (**d**) or in combination (**e**). Relative firefly luciferase activity of each condition was normalized to β-galactosidase activity and subtracted by basal luciferase activity obtained with the vector without consensus sequence of the NF-κB transcription factor. Values are mean ± SD from four independent experiments performed in duplicate and were compared by nonparametric Mann–Whitney U test (GraphPad Prism 6 software). *P < 0.05, ***P < 0.001.

To investigate whether TNFR1-d2 physically interacts with TNFR1, we performed immuno-precipitation experiments, in the absence or presence of TNFα (Fig. 4a). Our results indicate a direct interaction between both proteins, which does not seem to be modified by the presence of the ligand. A recent study revealed the importance of the TNFR1 organization at the plasma membrane for the activation of the downstream signalling cascades^33^. Using the Incucyte^®^ S3 Live-Cell Analysis System, we monitored in real-time the kinetic of assembly of TNFR1 and TNFR1-d2 clusters in our reporter system, in presence and absence of TNFα. In agreement with Morton et al.^33^, we found that TNFR1 formed preassembled clusters at the cell surface in the absence of the ligand (Fig. 4b). Our data revealed that the TNFR1-d2 was also able to form preassembled clusters at the cell surface without TNFα, although with a reduced speed compared to full-length TNFR1 (0.22 and 0.25 signal/min, respectively), leading to 20% less preassembled clusters than TNFR1 24 h post-transfection (Fig. 4b). This difference remains stable over time and the presence of TNFα did not change the number of TNFR1 and TNFR1-d2 clusters (Fig. 4c). These data suggest that, like TNFR1, TNFR1-d2 clustering might not be altered by TNFα ligand.

### Impact of the TNFR1-d2 isoform on the downstream signalling

To determine whether TNFR1-d2 plays a functional role in TNFR1 signalling pathways, we monitored the activation of NF-κB using a luciferase vector under the control of a NF-κB consensus promoter. As expected, TNFR1 dose-dependently activated NF-κB, unlike TNFR1-d2, as compared with the empty vector (Fig. 4d). When co-transfected with TNFR1, TNFR1-d2 had no effect on TNFR1-induced activation of NF-κB (Fig. 4e). These results reveal that TNFR1-d2 does not induce or interfere with NF-κB activation under normal conditions. Thus, the physical interaction between the TNFR1 full-length and TNFR1-d2 isoform might not be functionally meaningful in terms of NF-κB activation.

We quantified the expression levels of genes associated MAPK and NF-κB pathways, such as the MAPK p38 and MAPK1 (also named ERK2), and the Rel-like domain-containing protein RELA/p65 and NF-κB Essential Modulator (NEMO), respectively, as well as the TNFR1-associated death domain protein (TRADD), adaptor molecule that interacts with TNFR1 and mediates programmed cell death signaling and NF-κB activation. Upon TNFα stimulation, TNFR1 induced the mRNA expression of genes associated with NF-κB and MAPK pathways while TNFR1-d2 did not (Supplementary Fig. S5a). We thus simultaneously quantified the activation of canonical and non-canonical NF-κB subunits (p50, p52, p65, c-Rel, RelB) in nuclear extracts using a high throughput assay. Upon transfection with TNFR1 and TNFR1-d2, the non-canonical pathway components (p52 and RelB) were either not expressed or barely detectable. For the canonical pathway, only p65 expression showed differences between conditions (p50 and c-Rel did not), with an increase in cells over-expressing the TNFR1 (data not shown). Finally, using an AP-1 reporter assay, AP-1 activation was not detected following transfections with increasing doses of either TNFR1 or TNFR1-d2, in presence or absence of TNF stimulation (Supplementary Fig. S5b). These data suggest that, in our model system, only NF-κB p65 was activated upon TNFR1 engagement and that TNFR1-d2 neither activated NF-κB nor MAPK pathways.

### Alternative translation in TNFR1-d2 carrying the p.(Thr79Met) pathogenic variant

**Figure 5:**
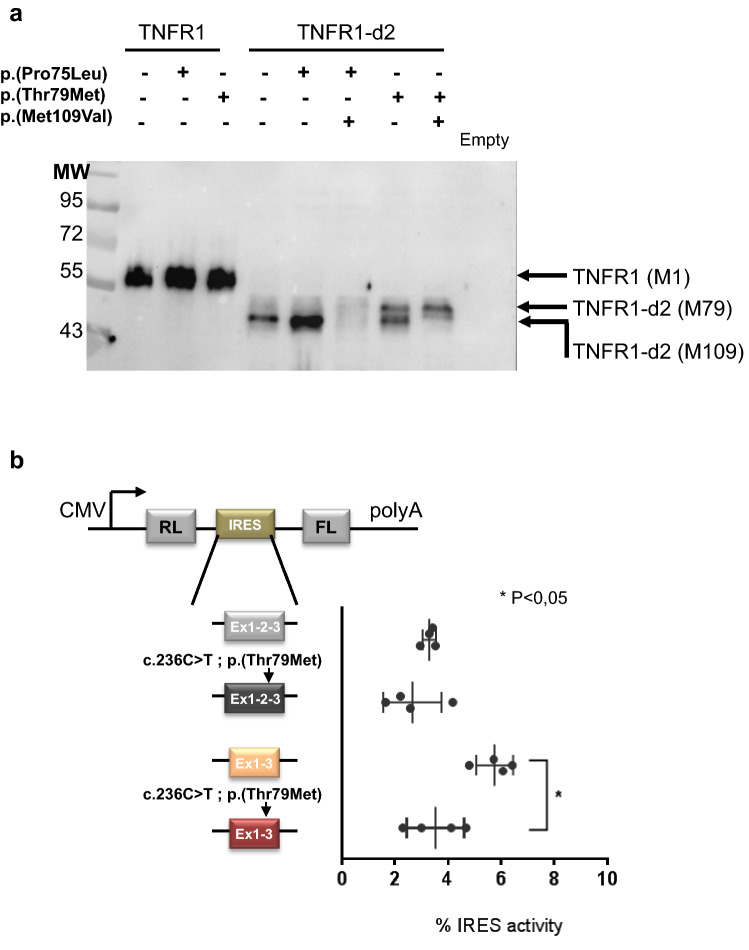
Translation defect of TNFR1-d2 carrying the c.236C>T pathogenic variant [p.(Thr79Met)]. (**a**) TNFR1 and TNFR1-d2 carrying or not the two variants in exon 3 (c.224C>T, p.(Pro75Leu), rs4149637 and c.236C>T, p.(Thr79Met), rs104895219) were transiently expressed in HEK293T cells. Protein extracts were resolved on 10% SDS-PAGE and then immunoblotted with anti-TNFR1 antibody. MW is the molecular weight marker in kiloDaltons (kDa). (**b**) Effect of c.236C>T variant on IRES activity of the exon 1 to 3 sequence of TNFR1 and exon 1 and 3 sequences of TNFR1-d2 in bicistronic luciferase assay. Values are mean ± SD from four independent experiments performed in duplicate and compared by nonparametric Mann–Whitney U test (GraphPad Prism 6 software). *P < 0.05.

The cap-independent translation of TNFR1-d2 raised the possibility of a translation defect of this transcript carrying a sequence variant identified in TRAPS patients. Examination of the sequence upstream of the initial translation codon of TNFR1-d2 in a TRAPS context revealed that only two known sequence variants [c.224C>T (p.(Pro75Leu)) and c.236C>T (p.(Thr79Met)) both in exon 3] presented a good context at the + 4 and − 3 positions as described by the classic Kozak consensus sequence (Supplementary Fig. S6)^34^. Using site-directed mutagenesis, we inserted these disease-causing variants in TNFR1 and TNFR1-d2 cDNA in-frame with the Flag tag. We then monitored their translation after transfection (Fig. 5a). As compared with the wild-type construct, neither TNFR1 carrying the p.(Pro75Leu) or p.(Thr79Met) pathogenic variant nor TNFR1-d2 carrying the p.(Pro75Leu) pathogenic variant showed a change in translation as compared with the wild type. In contrast, we detected two products for TNFR1-d2 carrying the p.(Thr79Met) disease-causing variant and one product for TNFR1-d2 carrying both p.(Thr79Met) disease-causing variant and p.(Met109Val) artificial variant. These results suggest that TNFR1-d2 can also be translated through the mutated codon in exon 3 corresponding to the p.(Thr79Met) variant. In agreement with defective translation of TNFR1-d2 carrying p.(Thr79Met), the percentage IRES activity was decreased with the bicistronic vector containing the junction of exons 1 and 3 and the c.236C>T variant as compared with the TNFR1-d2 wild type (Fig. 5b). Our results suggest that Met79 in mutated TNFR1-d2 can serve as the first amino acid allowing expression of a protein that contains 30 additional amino acids as compared with wild-type TNFR1-d2 (Supplementary Fig. S7).

### Functional consequences of the p.(Thr79Met) pathogenic variant on TNFR1-d2 isoform

**Figure 6:**
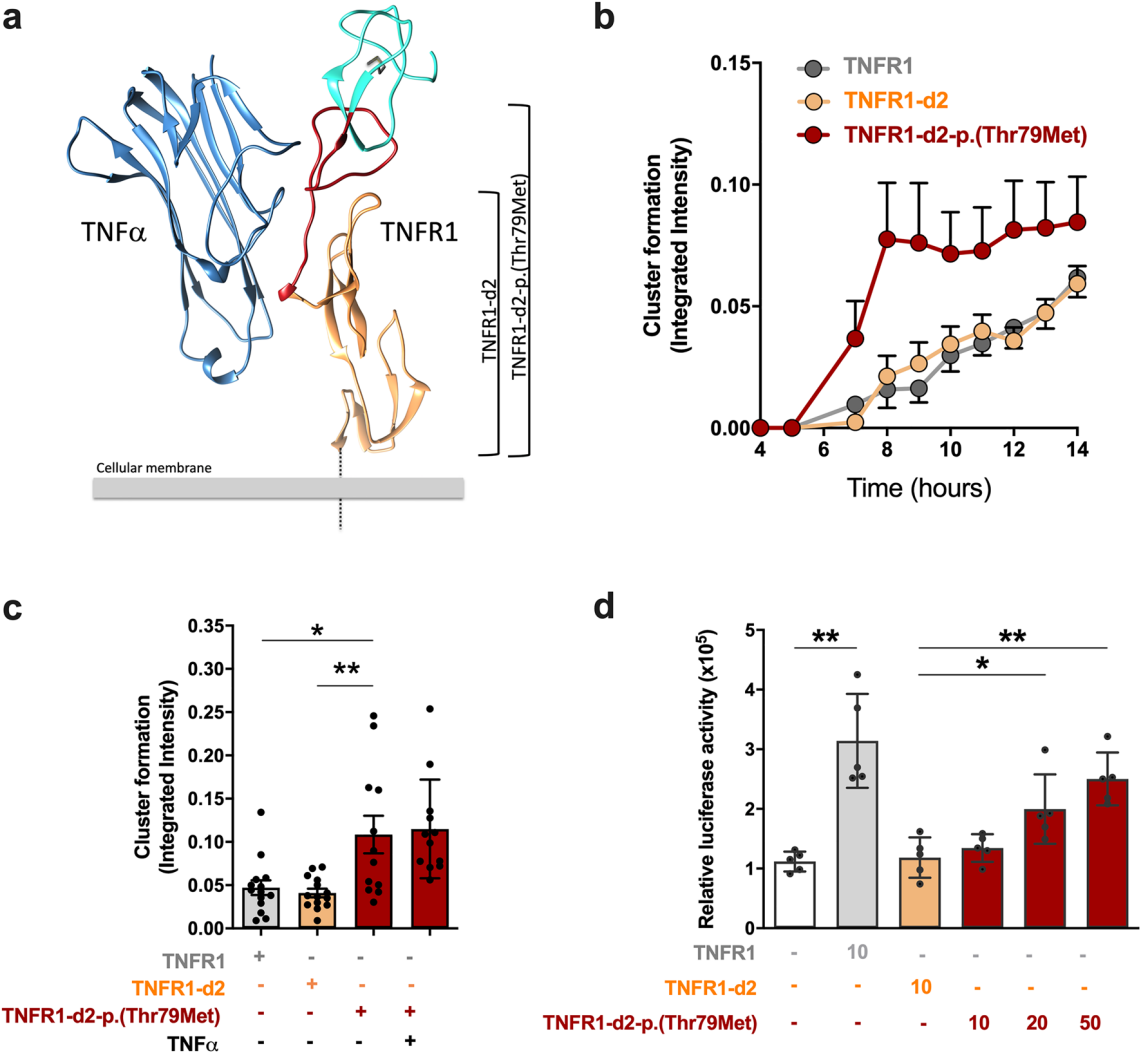
The p.(Thr79Met) pathogenic variant impacts TNFR1-d2 preassembled clusters formation and downstream NF-κB activation. (**a**) Representative structures of TNFR1, TNFR1-d2, TNFR1-d2-p.(Thr79Met) and TNFα. The TNFα ligand is in steel blue. The extracellular domain of TNFR1 is coloured to show TNFR1-d2 in sandy brown including CRD3 and CRD4 domains. TNFR1-d2-p.(Thr79Met) has additional CRD2 domain in red brick. The complete CRD1 domain is in turquoise. The chain of mutated residue M79 is shown in grey. (**b**,**c**) To determine the dynamics of receptor clusters oligomerization, HEK293T cells were transiently transfected with GFP plasmids of TNFR1, TNFR1-d2 and TNFR1-d2-p.(Thr79Met). Fluorescent and phase-contrast images were monitored using the IncuCyte live cell imaging system (Essen Bioscience). The normalized GFP Integrated intensity, was taken every ten minutes during next 2 hours after the cell seeding, following every one-hour during 14 hours (**b**). Histograms represent the GFP intensity of both transfection conditions at 12 hours (**c**). (**d**) HEK293T cells were transiently transfected with the indicated amount of TNFR1 or TNFR1-d2 carrying or not the p.(Thr79Met) disease-causing variant. Relative firefly luciferase activity of each condition was normalized to β-galactosidase activity and subtracted by basal luciferase activity obtained with the vector without consensus sequence of the NF-κB transcription factor. Values are mean ± SD from four independent experiments performed in duplicate and were compared by nonparametric Mann–Whitney U test (GraphPad Prism 6 software). *P < 0.05, **P < 0.01.

Chan et al.^5^ introduce the notion of pre-liganded assembly domain (PLAD) that corresponds to the CRD1 domain. This PLAD domain is essential to the receptor homotrimerization and to the stabilization of CRD2 domain conformation that is permissive for ligand-induced activation of TNFR1^33,35^. We first investigated the impact of the p.(Thr79Met) disease-causing variant on the TNFR1-d2 protein conformation using computational 3-D modelling from the structure of the complex formed by the extracellular domain of TNFR1 and TNFα (Fig. 6a). The conformation of the mutated TNFR1-d2 extracellular domain was longer than the one of the wild-type isoform. Although the sequence of p.(Thr79Met) TNFR1-d2 has no N-terminal CRD1 domain, it includes the CRD2, CRD3 and CRD4 domains of TNFR1, and 3 amino acids of the CRD1, whereas wild-type TNFR1-d2 only includes CRD3 and CRD4 domains of TNFR1, and 9 amino acids of the CRD2 (Supplementary Fig. S7b). Thus, as opposed to the wild-type TNFR1-d2 isoform, the elongated end of the mutated TNFR1-d2 isoform may be enough for TNFα binding.

We thus measured the kinetics of cluster formation at the cell surface, in absence and presence of TNFα. Our data showed that TNFR1-d2 carrying the p.(Thr79Met) disease-causing variant preassembled clusters at the cell surface in the absence of ligand more rapidly than wild-type TNFR1 and TNFR1-d2, reaching a plateau and twice more clusters formed than other proteins at 8 h post-transfection (Fig. 6b). This kinetic was not modified by addition of the ligand (Fig. 6c).

We then evaluated whether the p.(Thr79Met) disease-causing variant impacts the NF-κB activation capability by TNFR1-d2 isoform using our reporter system. The protein expression levels of the mutant and the wild-type TNFR1-d2 were quantified using western blot and immunofluorescent analyses (Supplementary Fig. S8). Although the mutant expression was less efficient than the wild-type one, the presence of the p.(Thr79Met) pathogenic variant in TNFR1-d2 significantly and dose-dependently increased spontaneous NF-κB activity as compared with the empty vector or wild-type TNFR1-d2 (Fig. 6d). When comparing conditions that gave the same levels of expression for both TNFR1 and TNFR1-d2-p.(Thr79Met) constructs, levels of NF-κB activation were comparable. Finally, the simultaneous quantification of the activation of canonical and non-canonical NF-κB subunits in nuclear extracts showed a significant and dose-dependent increase of p65 in cells over-expressing the TNFR1-d2 p.(Thr79Met) pathogenic variant (data not shown). Overall, our results suggest that the p.(Thr79Met) disease-causing variant conferred a gain of function to TNFR1-d2 that induces this isoform activate NF-κB signaling as efficiently as TNFR1.

## Discussion

Using in vitro models, we showed that the alternative TNFR1-d2 transcript of TNFR1 can be translated by an internal codon localized in exon 4, corresponding to Met109 in TNFR1. We also showed that the exon 1/exon 3 junction is involved in IRES activity. This novel TNFR1 isoform lacks CRD1 and most of CRD2, displays a close subcellular location with TNFR1, can interact with TNFR1 and is able to form preassembled clusters at the cell surface, but had no effect on NF-κB activation. Importantly, the p.(Thr79Met) pathogenic variant associated with TRAPS creates a novel translation initiation site and confers reduced IRES activity, leading to a TNFR1-d2 protein with elongated extracellular domain conformation able to preassemble clusters at the cell surface in the absence of ligand more rapidly than wild-type TNFR1 and TNFR1-d2, which induces spontaneous NF-κB activation.

TNFα, a pleiotropic cytokine, induces several complex signalling pathways via two receptors, TNFR1 and TNFR2. TNFR1 pathways lead to pro- or anti-survival cell-fate decisions, but TNFR2 has only a pro-inflammatory effect via NF-κB activation^1^. Recent studies showed a cross-talk between both pathways, which increases the complexity of the regulation of TNFα signalling^3,36^. Different levels of regulation have been revealed, including alternative splicing and translation, playing a role in protein diversity and functions^11^. Two TNFR2 alternative transcripts were identified in human. The splicing of exon 7 and 8 in the DS-TNFR2(Δ7,8) transcript directs the expression of a soluble form of TNFR2 lacking the transmembrane and cytoplasmic domains of TNFR2^37^. This isoform can be found in serum of healthy individuals, and high concentrations are associated with disease severity in rheumatoid arthritis^38^. Its level is also inversely correlated with metabolic disorders^39^ and liver injury^40^. The hicp75TNFR transcript, generated by an alternative transcriptional start site, leads to the expression of an intracellular receptor lacking the N-terminal part of TNFR2^41^. This isoform, mostly retained in trans-Golgi apparatus, activates NF-κB by binding to endogenous intracellular TNFα^42^. It is tempting to speculate on the existence of a parallel mechanism for TNFR1 regulation with the two Δ6-TNFR1 and TNFR1-d2 transcripts. The Δ6-TNFR1 leads to a soluble form of TNFR1 consisting only of CRD1 to CRD3 and half of CRD4^28^. Here, we showed that TNFR1-d2 could be translated from an internal initial codon leading to the expression of a protein lacking most of the N-terminal part of TNFR1 and presenting a punctuated intracellular location close to TNFR1.

Depending on the type of in vitro experiments performed and the cells used, several subcellular locations have been documented for TNFR1, with a predominant location in the Golgi apparatus^30^. The intracellular sequestration of TNFR1 may constitute a reserve of receptors, which, after stimulation, could be redirected to the membrane or excreted in the supernatant^32^. The C-terminal domain necessary for this localization is retained in TNFR1-d2, so a close intracellular location between the two isoforms is not surprising.

Our in silico conformational analysis suggests that TNFR1-d2 probably cannot bind TNFα. This is in agreement with the absence of the pre-ligand assembly binding domain (PLAD) formed by CRD1, which is essential for receptor homotrimerization and in the stabilization of CRD2 conformation, which is permissive for ligand-induced activation of downstream cascades^5^. Although the TNFR1-d2 isoform does not retain the signal peptide, western blot analyses show that it can interact with TNFR1 and form preassembled clusters at the cell surface, but less efficiently than full-length TNFR1. As shown by Morton et al.^33^, the receptor function of TNFR1 is strongly related to the dynamics of receptor clusters for oligomerization, and under favourable conditions, the NF-κB but not the JNK and p38 pathways could be activated by one TNFα/TNFR1 interaction. In agreement with this work, we found that TNFR1 forms preassembled clusters at the cell surface in the absence of TNFα, which number is not modified by TNFα stimulation. Our in vitro results exclude an effect of TNFR1-d2 on spontaneous NF-κB activation and on TNFR1-mediated NF-κB activation. This is in contrast to the hicp75TNFR isoform. However, we cannot rule out a potential indirect effect of TNFR1-d2 on TNFR1-mediated NF-κB activation via its influence on the balance between pro- and anti-survival cell fates. Given that the cell death pathway involves TNFR1 engulfment in cells and that we detected an intracellular location of TNFR1-d2, TNFR1-d2 may influence the signalling pathways under stress conditions inducing the cell death fate. In support of this hypothesis, our results showed that owing to exon 2 splicing in TNFR1-d2, the exon 1–3 junction can be involved in formation of an IRES sequence allowing the cap-independent translation of TNFR1-d2. Indeed, several studies have reported that IRES sequences allow for the translation of specific cellular mRNAs when cap-dependent translation is blocked, such as stress conditions (hypoxia, apoptosis, or amino acid deprivation) or during the cell cycle^43,44^. The presence of an IRES sequence and a cap-independent mechanism of translation was discovered in the 1990s, first in viruses^45,46^ and then in eukaryotes^47^. IRES-mediated translation allows for the expression of proteins involved in various cellular processes including proliferation, angiogenesis, apoptosis, and stem cell pluripotency, for genes such as fibroblast growth factor 2^48^, vascular endothelial growth factor A^49^, p53^50^ and octamer-binding transcription factor 4^51^, respectively. This alternative mechanism, combined or not with alternative transcription, allows for the expression of several proteins from a single gene, which can then display different functions, including negative control of the major protein. Moreover, IRES-containing transcripts are highly tissue-specific. This finding agrees with our previous report showing that TNFR1-d2 transcript expression is tissue-specific, whereas TNFR1 expression is ubiquitous^25^. We propose that the alternative translation of TNFR1-d2 might lead to the expression of a novel TNFR1 isoform acting on the TNFα-mediating death pathway directly under stressful conditions or indirectly via negative control of the TNFR1-survival pathway. Further experiments are needed to decipher the effect of TNFR1-d2 on the various TNFR1-mediating signalling pathways.

We previously showed that the splicing of TNFR1-d2 might be regulated by polymorphisms associated with the TRAPS phenotype, so here we investigated the involvement of the TNFR1-d2 protein in TRAPS pathogenesis. We first tested whether a TNFR1-d2 transcript carrying pathogenic variants found in TRAPS patients could drive a translational defect, possibly leading to abnormal function. The low-penetrance variant p.(Pro75Leu)^21^ does not affect the translation of TNFR1-d2, whereas the severe p.(Thr79Met) disease-causing variant allowed for translation via Met79, decreases IRES activity and induces spontaneous activation of NF-κB. This defective translation allowed for the expression of an isoform containing the full CRD2 domain and three amino acids of CRD1 in addition to TNFR1-d2 (Supplementary Fig. S7). Although only 3 amino acids of the PLAD are present, the presence of the p.(Thr79Met) disease-causing variant in TNFR1-d2 may stabilize CRD2 domain conformation by introducing a new possibility of di-sulfide bond creation and stabilize different conformation of receptor homotrimerization that is permissive for downstream activation^33,35^. The lack of CDR1 domain in TNFR1-d2-p.(Thr79Met) could also release constrains on CRD2, which is required for ligand binding. Here, we showed that TNFR1-d2 carrying the p.(Thr79Met) disease-causing variant preassembled clusters at the cell surface in the absence of TNFα more rapidly than TNFR1 and TNFR1-d2, that the kinetic is unchanged by TNFα stimulation, and that only the NF-κB p65 subunit level is increased downstream. Based on these data, we propose that the p.(Thr79Met) pathogenic variant induces a stabilized extracellular domain conformation that leads to a constitutive active form of the TNFR1-d2 isoform.

Numerous studies have been performed with an in vitro system or TRAPS patient cells to delineate the molecular defects underlying the TRAPS pathophysiology, and several hypotheses have been proposed, with some contradictory results depending on the system used^22,23^. However, to date, the TRAPS pathogenesis remains unclear and may be cell- and/or mutation-dependent. Most of these studies analysed the effect of the p.(Thr79Met) disease-causing variant, the most severe non-cysteine variant^52^. TRAPS patients carrying the p.(Thr79Met) disease-causing variant have a severe phenotype, with disease refractory to therapy such as infliximab^53^ or anakinra^54^. In addition, TRAPS cells carrying p.(Thr79Met) show reduced TNFR1 receptor shedding^20,23,55^, hyper-responsiveness to lipopolysaccharide^56–58^, inappropriate cellular stress leading to reactive oxygen species production^49,56,59^ and aberrant NF-κB activation. The defective TNFR1-mediated NF-κB activation seems to be subunit-specific because only the NF-κB p65 subunit level is increased in peripheral blood mononuclear cells (PBMCs) from patients, whereas the level of the basal or TNFα-induced NF-κB p50 subunit is comparable to that in PBMCs from healthy controls^55^. In addition, infliximab treatment of PBMCs induces an anti-apoptotic effect in p.(Thr79Met) PBMCs and an increased level of the NF-κB p65 and c-Rel subunit, which explains why TRAPS patients carrying this mutation are refractory to this anti-TNF therapy^53^. Here, our results showing a spontaneous NF-κB activation with TNFR1-d2 carrying the p.(Thr79Met) pathogenic variant suggest an involvement of the mutated TNFR1-d2 isoform in TRAPS patients carrying this variant that would need future investigations.

## Conclusions

Our results suggest that TNFR1-d2 could interact with TNFR1 without interfering on TNFR1-mediated pathways, and open new avenues to improve our understanding of TNFR1-mediated pathways. The alternative protein translated from the TNFR1-d2 isoform carrying the p.(Thr79Met) pathogenic variant alters TNFR1 and TNFR1-d2 signalling pathways, and this gain of function could account for the pathophysiology of TRAPS in these patients and could represent a target for the development of new therapies for patients with inflammatory diseases.

## Methods

### Cell culture and transfection

HEK293T and HeLa cell lines were grown in complete DMEM/F12 (1:1) medium containing 10% (v/v) fetal bovine serum, 2 mM l-glutamine and 100 U/ml penicillin/streptomycin. Absence of mycoplasma contamination was regularly monitored in both cell lines by using the MycoAlertTM Mycoplasma Detection Kit (Lonza) according to the manufacturer’s instructions. For all transfection experiments, cells were seeded into a 24- or 6-well plates depending on the experiment in complete DMEM medium without antibiotic and were transfected with 0.8 or 4 µg total cDNAs in OptiMEM reduced serum medium supplemented with 1.5 or 7.5 µl Lipofectamine 2000, respectively. Transfection efficiencies were 10–12% and 80–90% at 6 and 24 h post-transfection for all vectors, respectively. Conditions that provide similar expression levels of proteins were 1:2:4 as determined by fluorescence and western blot analyses of TNFR1, TNFR1-d2 and TNFR1-d2-p.(Thr79Met), respectively.

For HeLa cells, the medium was replaced by complete DMEM without antibiotic at 6 h post-transfection. Genetically modified organisms were declared at the national competent authority (agreement no. 2436).

### Plasmid construction

The pcDNA6/V5-His vector containing TNFR1 cDNA (NM_001065.3) was used to generate all TNFR1 vectors (a gift of the McDermott laboratory^59^). The TNFR1-d2 cDNA (GenBank accession no. JN172914^25^) was obtained by RT-PCR from human kidney total RNA (human Total RNA Master Panel II, Clontech). Vectors expressing TNFR1 and TNFR1-d2 cDNAs fused to Flag tag (Figs. 1b, 3, 4a,d,e and 5a), GFP or mCherry proteins (Figs. 2, 4b,c, 6b and supplemental Fig. S4) and bicistronic luciferase vectors (Figs. 1c and 5b) were constructed as described in Supplemental methods. Site-directed mutagenesis of c.1A>G [p.(Met1Val)], c.224C>T [p.(Pro75Leu)], c.236C>T [p.(Thr79Met)], and c.325A>G [p.(Met109Val)] was obtained by Pfu-mediated amplification with 5′ phosphate-modified and mispaired oligonucleotide primers described in supplemental Table, followed by a DpnI digestion. Sequence integrity of all constructs was confirmed by DNA sequencing.

### Western blot analysis and co-immunoprecipitations

HEK293T cells were seeded in 6-well plates at 4 × 10^5^ cells/ml before transfection or co-transfection with vectors expressing TNFR1 and/or TNFR1-d2 fused or not to Flag tag in presence and absence of TNFα (10 ng/ml). 48 h post-transfection, cells were lysed in 200 µl lysis buffer (CelLytic M, Sigma Aldrich) supplemented with 1X protease inhibitor cocktail (Roche Diagnostics) on ice for 20 min. After cold centrifugation at 13,000 rpm for 15 min, protein concentrations were determined using the BCA protein assay kit (Pierce). Adjusted amounts of protein extracts were immunopurified using an anti-FLAG M2 Affinity Gel (Sigma-Aldrich) according to the manufacturer’s instructions before western blot analysis (Figs. 1b and 4a) or were directly (Fig. 5a) examined by western blot analysis. Proteins were resolved on 10% SDS–polyacrylamide gel under reducing conditions and transferred to a nitrocellulose membrane except for co-immunoprecipitation experiments (Nupage 4–12% Bis–Tris Midi gel system). Membranes were blocked in TBS 1X containing 0.2% Tween (TBST) with 5% non-fat dry milk, then probed overnight at 4 °C with a 1:200 dilution of polyclonal goat anti-TNFR1 antibody directed against the C-terminal end of TNFR1 (TNF-R1(S-20), sc31350, Santa Cruz Biotechnology) in TBST supplemented with 3% non-fat dry milk. After a wash with TBST, a 1:5000 dilution of donkey anti-goat IgG-HRP secondary antibody (sc2020, Santa Cruz Biotechnology) was added for 1 h at room temperature. The blots were developed by using Supersignal West Pico chemiluminescent substrate (Pierce) with ChemiDoc XRS + (Bio-Rad) equipment and figures were created by using Image Lab 4.1.

### Bicistronic reporter assay

To evaluate IRES activity by bicistronic reporter assay, we used a series of bicistronic vectors containing the following intercistronic regions: *TNFRF1A* exons 1 to 3 or exon 1 and 3 sequences to assess TNFR1 or TNFR1-d2, respectively; the viral IRES sequence from encephalomyocarditis virus was a positive control (the pCREL vector^60^) or a hairpin sequence was a negative control (the pCRHL vector^57^). The two last vectors were a gift from the Prats laboratory. A 700 ng amount of these vectors was co-transfected in HEK293T cells seeded at 2 × 10^5^ c/ml in 24-well plates with 100 ng of the β-galactosidase vector (pCMV-LacZ, Clontech) used for internal normalisation for transfection efficiency. After 24 h, cells were collected with 200 µl of 1X Cell Culture Lysis reagent (Promega) and lysates were transferred to 96-well plates. After two freeze–thaw cycles at − 80 °C and 37 °C, cell debris were cleared by cold centrifugation at 4500 rpm for 10 min and protein concentrations were determined by using the BCA protein assay kit (Pierce) with 10 µl cleared lysates. To measure β-galactosidase activity, 20 µl cleared lysates were added to 140 µl buffer containing 60 mM Na_2_HPO_4_, 12H_2_O; 40 mM NaH_2_PO_4_; 0.34 mg/ml chlorophenol Red β-d-galactopyranoside and 2 mM MgCl_2_. After incubation for at least 30 min at 37 °C, β-galactosidase activity was determined by reading optical density at 570 nm.

A 20-µl amount of one-fifth dilution of cleared lysates was transferred to Microlite White Microtiter Plates, and the activity of Firefly luciferase (LucF) and Renilla luciferase (LucR) was determined by using the Dual-Luciferase Reporter Assay System (Promega), according to the manufacturer’s instructions. LucF and LucR were measured sequentially with double injection by using the Thermo Scientific Varioskan Flash Multimode Reader. LucF values were normalized by LucR values, β-galactosidase activity and protein concentration. The percentage IRES activity with each construct was calculated as a percentage of the ratio obtained with the positive control vector (the pCREL vector).

### Co-expression of TNFR1-mcherry and TNFR1-d2-GFP

HeLa cell lines were seeded on ethanol-treated glass coverslips in 24-well plates at 2 × 10^5^ cells/ml and transfected with 800 ng vectors encoding mCherry and GFP proteins alone or fused in the C-terminal end of TNFR1 or the coding sequence of TNFR1-d2, respectively. Co-localization of TNFR1 and TNFR1-d2 was analysed by co-transfection of 400 ng TNFR1-mCherry and 400 ng TNFR1-d2-GFP vectors. After 24 h post-transfection, cells were washed with phosphate-buffered saline (PBS) and fixed in 4% paraformaldehyde in PBS for 10 min. After three washes of 5 min in PBS, cells were analysed by confocal microscopy, as described in the section fluorescence microscopy analysis.

### Immunofluorescent staining

HeLa cell lines were seeded on ethanol-treated glass coverslips in 24-well plates at 2 × 10^5^ cells/ml before transfection with 800 ng vectors expressing TNFR1 proteins fused to C-terminal Flag tag. For this experiment, we used a TNFR1-d2 vector containing the coding sequence of TNFR1-d2 (from A^171^TG to exon 10, Fig. 1a). After 24 h post-transfection, cells were washed with PBS and fixed in 4% paraformaldehyde in PBS for 10 min. After three washes of 5 min in PBS, cells were permeabilized with 0.5% Triton X-100 in PBS for 5 min. Then, cells were washed three times with PBS, blocked for 1 h in PBS containing 5% bovine serum albumin for co-staining membrane junctions and mitochondria (Fig. 3a,b) or 10% normal donkey serum (NDS, Abcam) for co-staining endoplasmic reticulum and the Golgi apparatus (Fig. 3c,d). All labelling is described in Supplemental methods.

### Fluorescence microscopy analysis

After co-expression of TNFR1-mcherry and TNFR1-d2-GFP (Fig. 2) and immunofluorescence staining (Fig. 3), cells were mounted on slides with 10 µl ProLong Diamond Antifade Mountant with DAPI (Molecular Probes) for nuclei counterstaining. Fluorescent cells were observed under a Zeiss Leica TCS SP5 inverted confocal laser-scanning microscope with the appropriate filters and laser and a 63× objective. Pictures were acquired with the Leica LAS AF software and representative merged images and figures were created by using ImageJ. At least 15 random fields for each experiment were acquired and the co-localization index was calculated by using M1 and M2 Manders’ coefficients with the plugin JACoP (Just Another Co-localization Plugin^29^) of ImageJ. The M1 coefficient measures the fraction of the green channel overlapping the red channel and the M2 coefficient the fraction of the red channel overlapping the green channel. Ranging from 0 to 1, the Manders’ coefficients allowed to differentiate no, occasional, partial or complete co-localization with the two channels.

### IncuCyte kinetic monitoring of receptor clusters oligomerization

To determine the dynamics of receptor clusters oligomerization, we plated 5 × 10^4^ HEK293T cells per well in 24-well plates and cultured them in complete DMEM/F12. Cells were transfected with GFP plasmids of TNFR1, TNFR1-d2 and TNFR1-d2-p.(Thr79Met), and immediately stimulated or not with TNFα (10 ng/ml) and monitored using the Incucyte^®^ S3 Live-Cell Analysis System, 20× magnification (Essen is now part of Sartorius (MI, USA)). Following transfection, fluorescent and phase-contrast images were taken every ten minutes during 2 h, and then every hour during 48 h. Experiments were performed in triplicates for each transfection conditions. Fluorescent and phase-contrast images were analyzed with the Incucyte Analysis Software. Green Integrated Intensity was normalized with total green object Area (transfected cells) to obtain cluster formation rate.

### Spontaneous NF-κB activation

HEK293T cells seeded in 24-well plates at 4 × 10^5^ cells/ml were transfected with a suitable amount of vectors expressing TNFR1 or TNFR1-d2 fused to Flag tag (from 10 to 70 ng) along with 10 ng β-galactosidase vector and 50 ng of a vector containing luciferase firefly cDNA under the control of a constitutive promoter region combined or not with four copies of the NF-kB consensus sequence (pNFkB-luc or pTAL-luc, respectively, Clontech). Total amount of transfected vector (800 ng) was obtained by the addition of an appropriate amount of empty pcDNA vector. After 24 h, cells were harvested and β-galactosidase activity was measured in 20 µl cleared lysates as described in the bicistronic reporter assay section. By using the Thermo Scientific Varioskan Flash Multimode Reader, firefly luciferase activity was measured on 50 µl cleared lysates diluted five times after a 50-µl single injection of buffer containing 530 µM ATP (Sigma Aldrich), 470 µM luciferin (Sigma Aldrich), and 270 µM coenzyme A (Sigma Aldrich) in luciferase buffer [40 mM Tricine, pH 7.8; 2.14 mM (MgCO_3_); 4 Mg(OH)_2_, 5H_2_O; 5.34 mM MgSO_4_; 0.2 mM EDTA]. The relative firefly luciferase activity for each condition was normalized to β-galactosidase activity and was subtracted by the basal luciferase activity obtained with the pTAL-luc vector.

### 3-D modelling

The 3-D model conformational analysis of TNFR1-d2 was performed using computational modelling of the X-ray crystal structure of the complex formed by the extracellular domain of human TNFR1 and TNFα (1TNR PDB number) elucidated by Banner et al.^61^. The Chimera software^62^ was used to visualize and introduce the human disease-causing variant p.(Thr79Met) in the TNFR1-d2 structure.

### Statistical analysis

Each experiment was performed from 2 to 5 times depending on experiments and sometimes performed in duplicate (see legends of figures). Statistical significance was calculated by using the non-parametric Mann–Whitney U test to compare two groups and was assumed at p < 0.05 (GraphPad Prism 6).

## Supplementary Information

Supplementary Information.

## Data Availability

All data generated or analysed during this study are included in this published article or available from the corresponding author on reasonable request.
