# Supplementary material for: TNFR1-d2 carrying the p.(Thr79Met) pathogenic variant is a potential novel actor of TNFα/TNFR1 signalling regulation in the pathophysiology of TRAPS
Source: Sci Rep. 2021 Feb 18;11:4172. doi: 10.1038/s41598-021-83539-9 (PMC7893027; doi:10.1038/s41598-021-83539-9)
Supplement: Supplementary file 1 — Supplementary Information. [file 41598_2021_83539_MOESM1_ESM.docx]

**TNFR1-d2 carrying the** **p.(Thr79Met) pathogenic variant is a potential novel actor of TNFα/TNFR1 signalling regulation in the pathophysiology of TRAPS**

Cécile Rittore^1,2,§^, Déborah Méchin^1,2,§^, Elodie Sanchez^1,2^, Léa Marinèche^1^, Vuthy Ea^1^, Stephan Soler^1,2^, Marion Vereecke^1,2^, Aude Mallavialle^3^, Eric Richard^3^, Isabelle Duroux-Richard^1^, Florence Apparailly^1,4*^, Isabelle Touitou^1,2^, Sylvie Grandemange^1,2^

^1^ IRMB, INSERM, Univ Montpellier, Montpellier, France

^2^ CHU Montpellier, Department of Medical Genetics, Rare Diseases and Personalized Medicine, Montpellier, France

^3^ IRCM, INSERM, Univ Montpellier, Montpellier, France

^4^ CHU Montpellier, Clinical department for osteoarticular diseases, Univ Montpellier, Montpellier, France

^§^Authors equally contributed to this work

*Corresponding author: Florence Apparailly, INSERM U1183, CHU Saint Eloi, IRMB, 80 Avenue Augustin Fliche, 34295 Montpellier cedex 5, France

Tel: 33 467 335 696

Fax: 33 467 330 113

**Methods**

**Cloning of TNFR1-d2**

To clone the TNFR1-d2 cDNA (GenBank accession no. JN172914[1]), we used RT-PCR analysis of human kidney total RNA (human Total RNA Master Panel II, Clontech). RNA was reverse-transcribed into cDNA by using Moloney Murine Leukemia Virus Reverse Transcriptase and random primers. The TNFR1-d2 cDNA was amplified with high-fidelity Pfu DNA polymerase with the forward primer in exon 1 (5’- CCGATATCATGGGCCTCTCCACCGTG-3’) and reverse primer in exon 10 (5’- CCGCTCGAGTCTGAGAAGACTGGGCG-3’). The PCR products were directly cloned into the pCR 2.1-TOPO TA vector by using the TOPO TA subcloning kits according to the manufacturer’s instructions (Invitrogen). This vector was used to generate the TNFR1-d2-Flag vector.

**Flag expressing vectors**

The pcDNA6/V5-His-TNFR1 vector (NM_001065.3, a gift of the McDermott laboratory[2]) was used as a template to amplify TNFR1 cDNA. TNFR1 was amplified with high-fidelity Pfu DNA polymerase (Promega) with the forward primer in exon 1 (5’- CCGATATCATGGGCCTCTCCACCGTG-3’) and reverse primer in exon 10 (5’- CCGCTCGAGTCTGAGAAGACTGGGCG-3’) containing the EcoRV and XhoI restriction enzyme sites, respectively (underlined). PCR product was migrated on agarose gel and the band corresponding to the full-length TNFR1 cDNA was purified by using the Invitrogen PureLink Quick Gel Extraction Kit. After appropriate enzyme digestion, the insert was ligated into the corresponding sites of the pCMV-Tag4B (Agilent Technologies) in the C-terminal frame of the Flag tag. This vector was used in overexpression experiments, subcellular localization and NF-κB assays (Fig. 1b, Fig. 2, Fig. 4, Fig. 5a and c).

The TNFR1-d2 cDNA (GenBank accession no JN172914[1]) in the pCR 2.1-TOPO TA vector was subcloned in frame in the Flag tag by digestion with the EcoRV and XhoI enzymes. The vector expressing the full-length of TNFR1-d2 fused to Flag tag was used in overexpression experiments and in the NF-κB luciferase assay (Fig. 1b, Fig. 4, Fig. 5a and c) and was used as a template to generate the TNFR1-d2 vector containing only the coding sequence of TNFR1-d2 (from A^171^TG to exon 10). After Pfu amplification with the forward primer in exon 4 (5’- CCGATATCATGGGTCAGGTGGAGATC -3’) and reverse primer in exon 10 (5’- CCGCTCGAGTCTGAGAAGACTGGGCG-3’) containing sites for the EcoRV and XhoI restriction enzymes, respectively (underlined), the digested fragment was subcloned in the pCMV-Tag4B in the C-terminal frame of the Flag tag. This vector was used in subcellular localization experiments (Fig. 2).

**Plasmid construction for confocal microscopy analysis**

To create the vector expressing TNFR1 and TNFR1-d2 fused in C-terminal with GFP, we used as a template the pCMV-Tag4B containing the TNFR1 and the coding sequence of TNFR1-d2. These vectors were obtained by Pfu-mediated amplification with forward 5’- CGCGGATCCATGGGCCTCTCCACCGTG -3’ and reverse 5’- CGCGGATCCTCTGAGAAGACTGGGCG -3’ primer sequences containing the BamHI restriction enzyme sites (underlined). Amplification products were cut with BamHI and inserted in the pcDNA3 vector containing GFP protein from the jellyfish *Aequorea victoria*. These vectors were used in co-localization experiments with the Golgi apparatus (Supplementary Fig. S4) and in co-localization assay for TNFR1-d2 (Fig. 3).

To generate vectors expressing TNFR1 fused to mCherry protein used in co-localization assays (Fig. 3), we amplified TNFR1 cDNA from the TNFR1-GFP vector with Pfu DNA polymerase and forward and reverse primers (5’- CCGCTCGAGATGGGCCTCTCCACCGTGC-3’ and 5’- CGCGGATCCTCGAGAAGACTGGGCGCGG-3’, respectively). After agarose gel purification, the TNFR1 insert was cut with XhoI and BamHI enzymes and inserted in frame in mCherry protein derived from *Discosoma sp. DsRed* (sea anemones) in the pmCherry-N1 vector (Clontech).

**Bicistronic constructs for IRES assay**

To analyse IRES activity, we used a trimolecular strategy described in Martineau et al.[3] to construct vectors containing an intercistronic region containing exons 1, 2 and 3 of TNFR1 or exons 1 and 3 of TNFR1-d2, surrounding an upstream Renilla luciferase (RL) and a downstream Firefly luciferase (FL) cistron under control of the CMV promoter.

The CMV-RL fragment was derived from the pCR-F1AL vector described in Créancier et al.[4] by digesting the plasmid with the BglII and SpeI enzymes followed by agarose gel purification (a gift of the Prats laboratory).

The FL fragment was obtained by digestion with the NcoI and BglII enzymes of the pCREL vector[5] followed by agarose gel purification.

To generate inserts, we used the corresponding Flag vectors as a template and amplified exons 1, 2 and 3 of TNFR1 or exons 1 and 3 of TNFR1-d2 by using AmpliTaq Gold DNA Polymerase (Applied Biosystems) and a forward primer in exon 1 (5’-ACTAGTATGGGCCTCTCCACCGTG-3’) and a reverse primer in exon 3 (5’-CCATGGTTCCTTTCGGCATTTG-3’) containing the SpeI and NcoI restriction enzyme sites, respectively (underlined). The PCR products were purified on agarose gel and digested with SpeI and NcoI enzymes.

The three fragments were ligated by using quick ligase, and fidelity and orientation of the constructs was confirmed by DNA sequencing.

**Bodipy staining**

HeLa cell lines were seeded on ethanol-treated glass coverslips in 24-well plates at 2.10^5^ cells/ml before transfection of 800 ng vectors expressing TNFR1 proteins fused to GFP protein. After 24 h post-transfection, cells were incubated for 30 min at 4 °C with 5 μM BODIPY TR ceramide (Molecular Probes) in HBSS buffer containing 10 mM HEPES, followed by two washes with ice-cold PBS and incubation in fresh complete DMEM medium for 30 min at 37°C. Then, cells were washed with PBS and fixed in 4% paraformaldehyde in PBS for 10 min. After three washes of 5 min in PBS, cells were mounted on slides with 10 µl ProLong Diamond Antifade Mountant with DAPI (Molecular Probes) for nuclei counterstaining. Fluorescent cells were observed under a Zeiss Leica TCS SP5 inverted confocal laser-scanning microscope with the appropriate filters and laser and a 63× objective.

**Immunofluorescent staining**

HeLa cell lines were transfected with 800 ng vectors expressing TNFR1 proteins fused to C-terminal Flag Tag. After 24 h post-transfection, cells were washed, fixed and permeabilized with 0.5% Triton X-100 in PBS for 5 min, then blocked for 1 hr in PBS containing 5% bovine serum albumin (BSA; Fig. 3a and b) or 10% normal donkey serum (NDS, Abcam) (Fig. 3c and d). For all labelling, cells were incubated with appropriate primary antibodies overnight at 4°C in PBS containing 1% BSA or 1% NDS. After five washes with PBS supplemented with 0.05% Tween20, cells were stained with suitable fluorophore-conjugated secondary antibodies in the dark for 1 h at room temperature in PBS containing 1% BSA or 1% NDS. After washing, immunofluorescence staining was analysed by confocal microscopy as described in the fluorescence microscopy analysis section. TNFR1 and TNFR1-d2 proteins were stained with a FLAG® M2 monoclonal antibody (1:200, Sigma Aldrich) and an Alexa Fluor 555-conjugated goat anti-mouse IgG antibody (1:500, A21424 ThermoFisher) for co-staining of membrane junctions and mitochondria or an Alexa Fluor 555-conjugated donkey anti-mouse IgG antibody (1:500, A31570 ThermoFisher) for co-staining of endoplasmic reticulum or Golgi apparatus. The membrane junctions and mitochondria were stained with a rabbit antibody directed against β-catenin (1:100; 9562 Cell Signalling Technology) or Tom20 (1:500; sc11415 Santa Cruz Biotechnology), respectively, and an Alexa Fluor 488-conjugated goat anti-rabbit IgG antibody (1:500, A11008 ThermoFisher) in PBS containing 1% BSA. Co-staining of the endoplasmic reticulum or Golgi apparatus involved using a rabbit antibody for GRP94 (1:20; sc11402) or TGN38 (1:250; sc33783, both Santa Cruz Biotechnology) and an Alexa Fluor 488-conjugated donkey anti-rabbit IgG antibody (1:500, A21206 ThermoFisher) in PBS containing 1% NDS.

**AP-1 reporter assay**

HEK293T cells were seeded into 24 plates in complete DMEM medium without antibiotics and were co-transfected with 50 and 10 ng of pAP-1 (-517)-LucF vector provided by M. Mathieu (INSERM, Montpellier) and CMV-beta-Gal (Sigma), respectively, together with increasing amounts of TNFR1 or TNFR1-d2 encoding vector, in OptiMEM reduced serum medium supplemented with 1.5 µl Lipofectamine 2000. AP-1 luciferase and β-galactosidase activity were measured 24 hours post transfection, according to Mathieu et al[6] and the manufacturer’s recommendations, respectively. The β-galactosidase activity was measured to correct for differences in transfection efficiency and the luciferase activity for normalisation.

**Supplemental Table: Primers used for directed mutagenesis**

| **Insert** | **Vector** | **Sequence variant** | | **Primers** |
| --- | --- | --- | --- | --- |
| TNFR1-d2 | pcDNA-Flag | c.1A>G | p.(Met1Val) | F: GGAATTCGATATCGTGGGCCTCTCCACCG |
|  |  |  |  | R: CGGTGGAGAGGCCCACGATATCGAATTCC |
| TNFR1 | pcDNA-Flag | c.224C>T | p.(Pro75Leu) | F: CTGTCCAGGCCTGGGGCAGGATACGG |
| TNFR1-d2 |  |  |  | R: CCGTATCCTGCCCCAGGCCTGGACAG |
| TNFR1 | pcDNA-Flag | c.236C>T | p.(Thr79Met) | F: CCCGGGGCAGGATATGGACTGCAGGGAGTG |
| TNFR1-d2 |  |  |  | R: CACTCCCTGCAGTCCATATCCTGCCCCGGG |
| ex1-ex2-ex3 | bicistronic | c.236C>T | p.(Thr79Met) | F: CCCGGGGCAGGATATGGACTGCAGGGAGTG |
| ex1-ex3 |  |  |  | R: CACTCCCTGCAGTCCATATCCTGCCCCGGG |
| TNFR1-d2 | pcDNA-Flag | c.325A>G | p.(Met109Val) | F: CCAAATGCCGAAAGGAAGTGGGTCAGGTGGAGATCTC |
|  |  |  |  | R: GAGATCTCCACCTGACCCACTTCCTTTCGGCATTTGG |

Ex, F and R for exon, forward and reverse respectively

Sequence variant is in TNFR1 nomenclature (NM_001065.3).

All primers were purchased from MWG.

**Supplemental Figure legends**

**Fig. S1 Open reading frames of TNFR1-d2 transcript**

**a.** The sequences encoding the signal peptide, cysteine-rich domain and death domain of the TNFR1 protein are depicted in blue, orange, and green letters, respectively.

**b**. Putative amino acid sequences of TNFR1-d2 translated in the three frames. The same colour code as for TNFR1 is used. All methionines that can be a potential translation start codon are in bold. Asterisks represent stop codons. M^1^ corresponds to the first methionine (A^1^TG) in the TNFR1 nomenclature. M^109^ delineates the potential initial codon of TNFR1-d2 (A^325^TG or A^171^TG in the TNFR1 or TNFR1-d2 nomenclatures, respectively).

**Fig. S2. Occasional co-localization of TNFR1-d2 with membrane junction and mitochondria**

Non-merged representative images of subcellular co-localization. Panel a (Fig. 3a) and panel b (Fig. 3b).

**Fig. S3. Partial co-localization of TNFR1-d2 in endoplasmic reticulum**

Non-merged representative images of subcellular co-localization. Panel a (Fig. 3c) and panel b (Fig. 3d).

**Fig. S4. Co-localization of TNFR1 and TNFR1-d2 with Golgi apparatus.**

TNFR1 or TNFR1-d2 fused to C-terminal GFP proteins were transfected in HeLa cells. After 24 h, cells were stained with Bodipy Golgi tracker, counterstained with DAPI and analysed by confocal microscopy. Representative zoomed images of one independent experiment are shown. On the right, the boxes correspond to the median and minimal and maximum co-localization index of TNFR1 and TNFR1-d2 with the Golgi apparatus. At least 5 random fields were analysed and co-localization index was calculated by using Manders’ coefficients that represent the fraction of TNFR1 or TNFR1-d2 (green) overlapping the red channel (JaCoP plugin in ImageJ software).

**Fig. S5. Monitoring of the NF-κB and MAPK signalling pathways.**

HEK293T cells were transiently transfected with vectors containing either TNFR1 (0.8µg) or TNFR1-d2 (1.5µg), over 24 hours, with or without TNF stimulation (10µg/ml). (**a**) Following RNA extraction, genes associated with MAPK and NF-κB pathways, such as P38, ERK2 and REAL, NEMO, respectively, as well as TRADD, an adaptor molecule that interacts with TNFR1 and mediates programmed cell death signalling and NF-κB activation, were quantified using RT-qPCR. Data are presented as fold change between stimulated and unstimulated conditions. (**b**) HEK293T cells were co-transfected with a vector encoding an AP-1 luciferase reporter gene, along with increasing amounts of TNFR1 or TNFR1-d2 plasmids. AP-1 activity was measured using luciferase assay 24 hours post-transfection (n=4).

**Fig. S6. Sequences around the p.(Pro75Leu) and p.(Thr79Met) TRAPS disease causing variants are close to a Kozak consensus sequence**

The four codons located upstream of the initial codon of TNFR1-d2 (p.M109) and potentially encoding a start codon in the TRAPS context are represented in TNFR1 nomenclature. Blue and red colours denote the wild-type and TRAPS-mutated sequences, respectively. The putative Kozak sequences around the 4 codons are depicted on the top. The black bold letters show the crucial +4 and -3 positions as described for the classic Kozak consensus sequence depicted below.

**Fig. S7. Schematic representation of TNFR1-d2 wild type and TNFR1-d2- p.(Thr79Met)**

(**a-b**) Schematic representations of TNFR1 and TNFR1-d2 transcripts (**a**) and proteins (**b**). Location of the first codon in exon 1 and the initial codon for TNFR1-d2 carrying or not the p.(Thr79Met) pathogenic variant are indicated by arrows in the TNFR1 nomenclature. The same colours in transcripts and in proteins were used to depict the domains of TNFR1 protein. aa: amino acids, PLAD: pre-liganded assembly domain and CRD: cystein rich domain.

**Fig. S8. TNRF1, TNFR1-d2 and TNFR1-d2-** **d2-p.(Thr79Met) expression levels.** (**a**) HEK293T cells were transiently transfected with GFP plasmids of TNFR1, TNFR1-d2 or TNFR1-d2-p.(Thr79Met). Fluorescent and phase-contrast images were measured using the IncuCyte live cell imaging system (Essen Bioscience). Histograms represent the GFP intensity monitored on 13 different areas at 6 and 24 hours post-transfection. (**b**) HEK293T cells were transiently transfected with the indicated doses of vectors expressing either TNFR1-d2 or TNFR1-d2-p.(Thr79Met). Western blot analysis was performed using anti-flag antibody and quantified with ImageLab software (Biorad). Protein expression was normalized with actin expression levels.

**
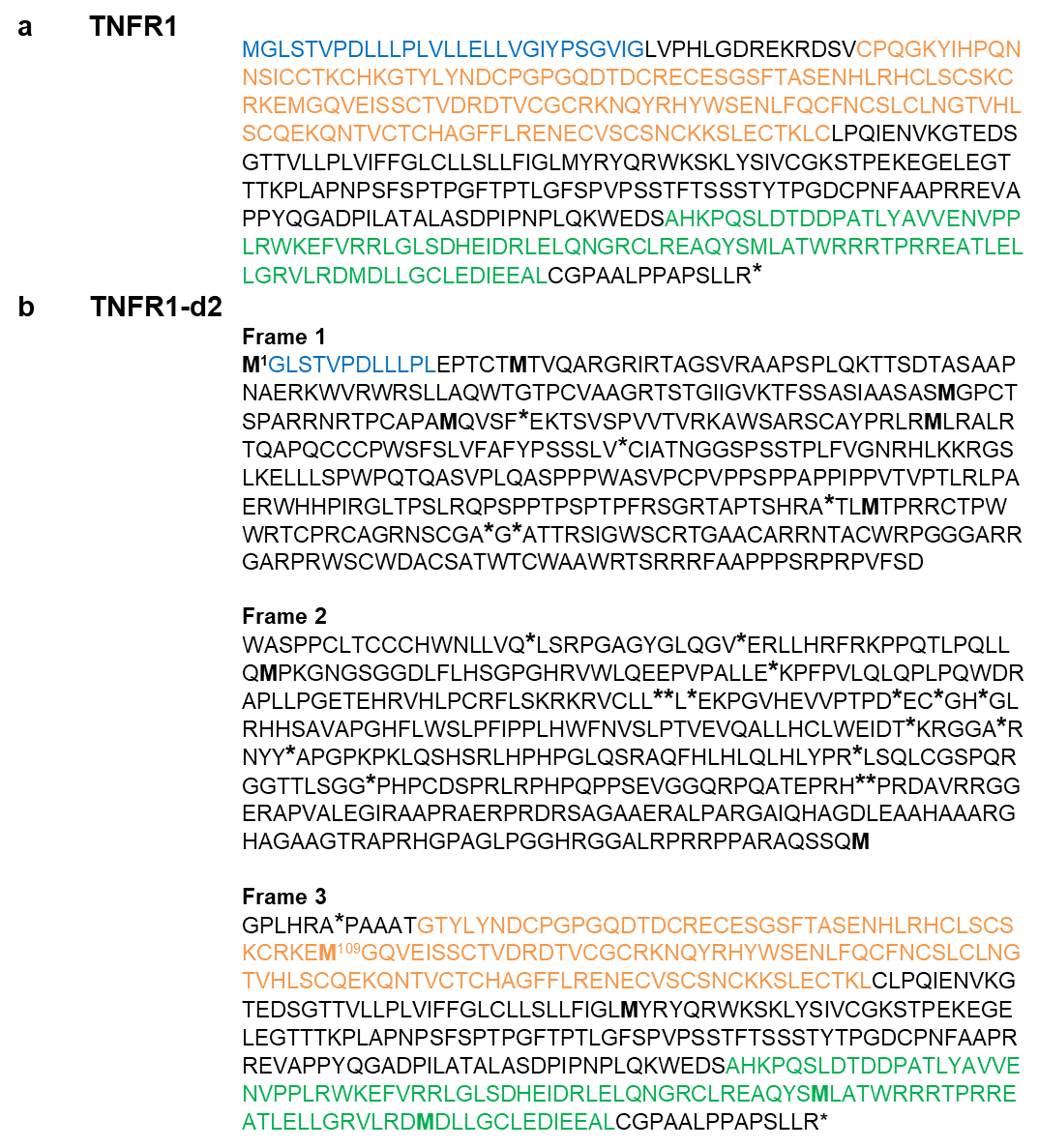
**

**Supplementary Fig. S2**

**
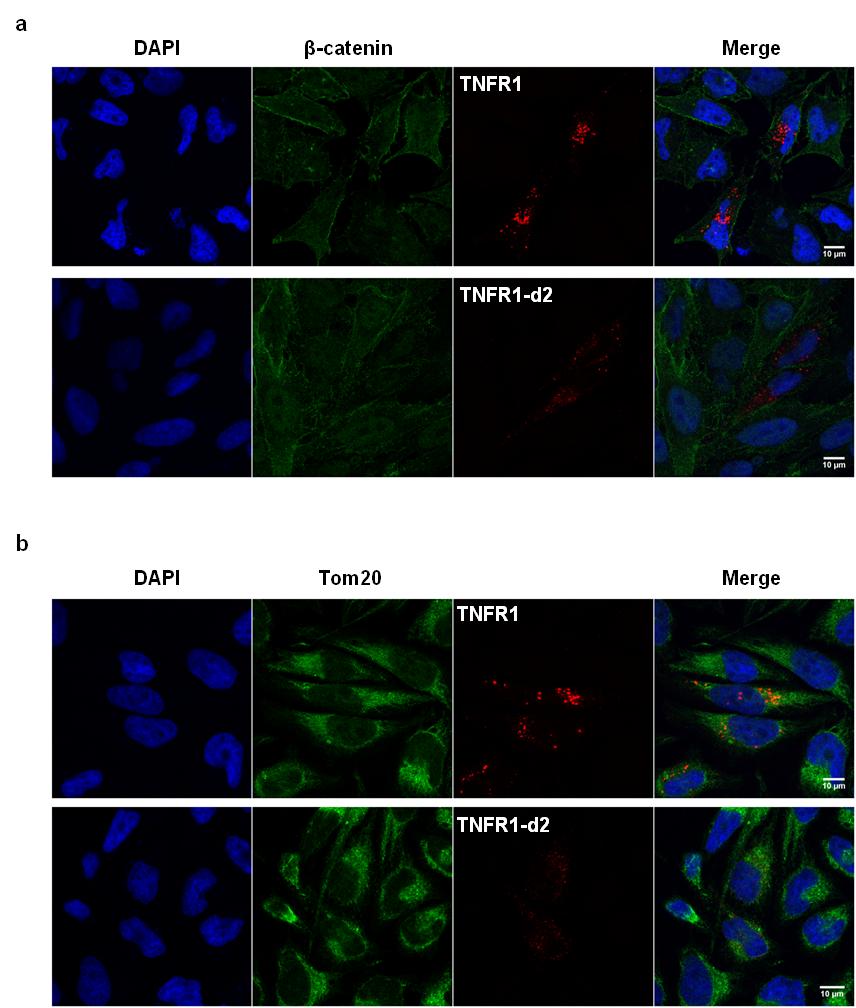
**

**Supplementary Fig. S3**

**
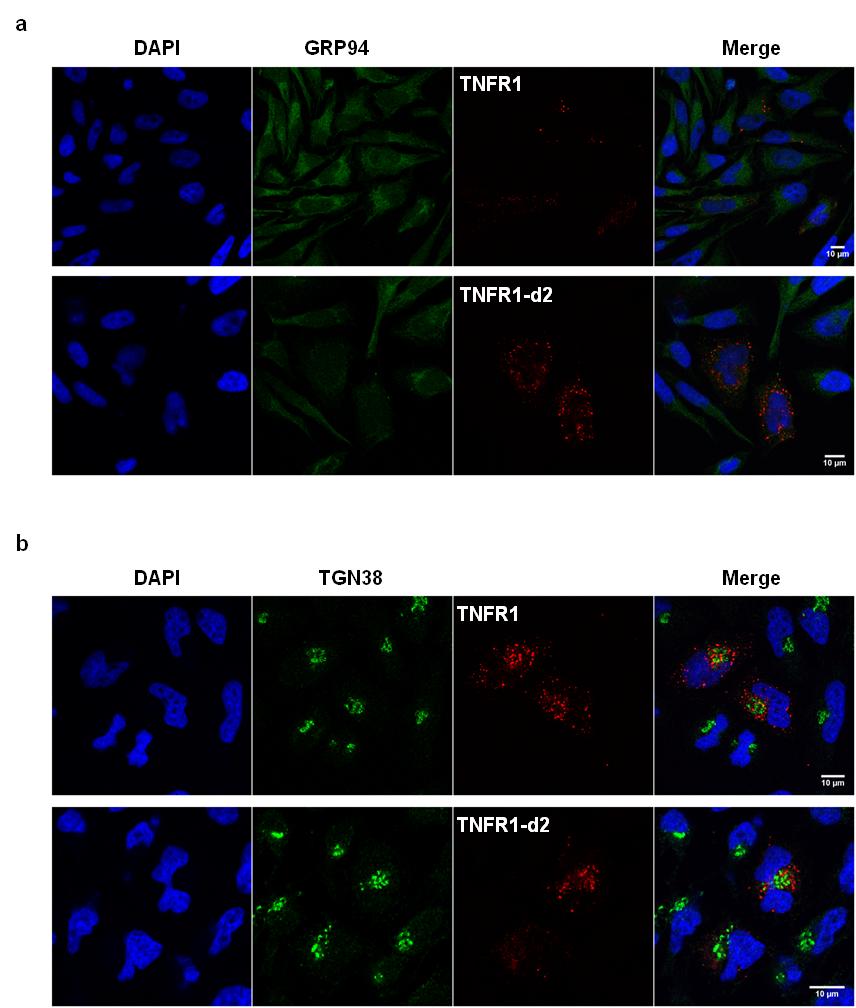
**

**Supplementary Fig. S4**

**
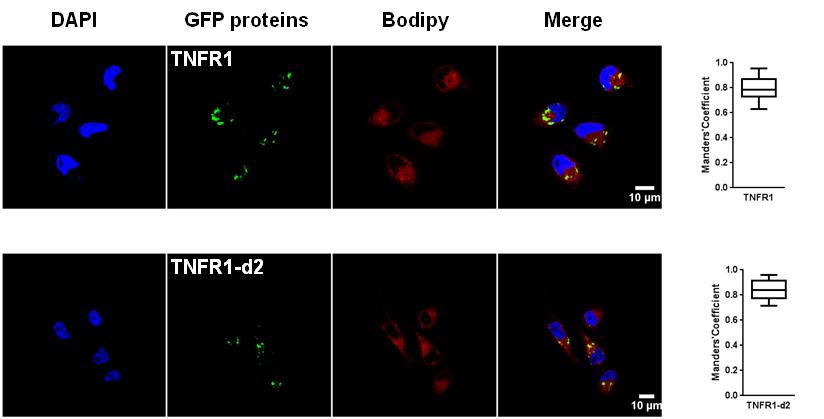
**

**Supplementary Fig. S5
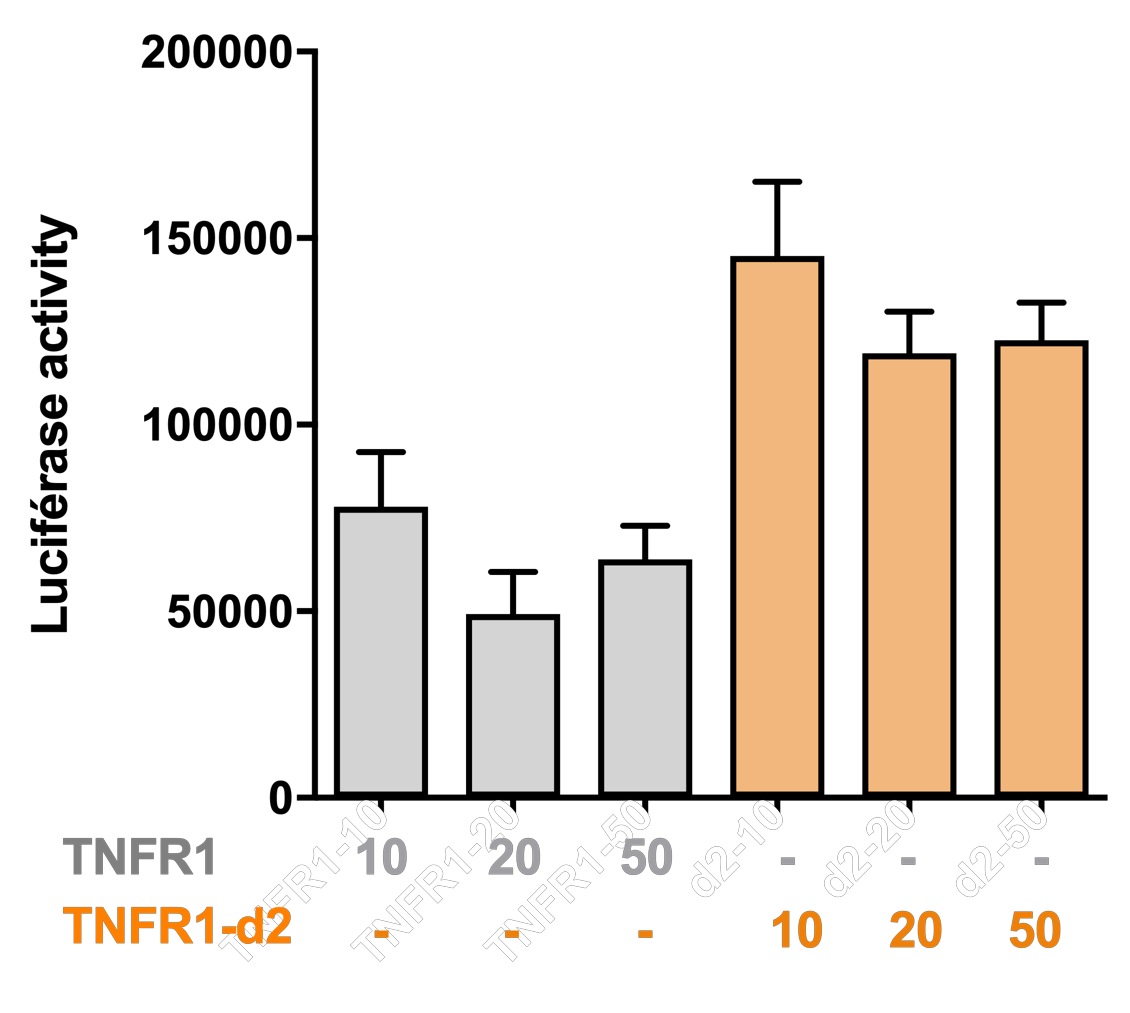
**

**a**

**b**

MAPK Pathway

NF-κB Pathway

AP-1 activity

**Supplementary Fig. S6**

**
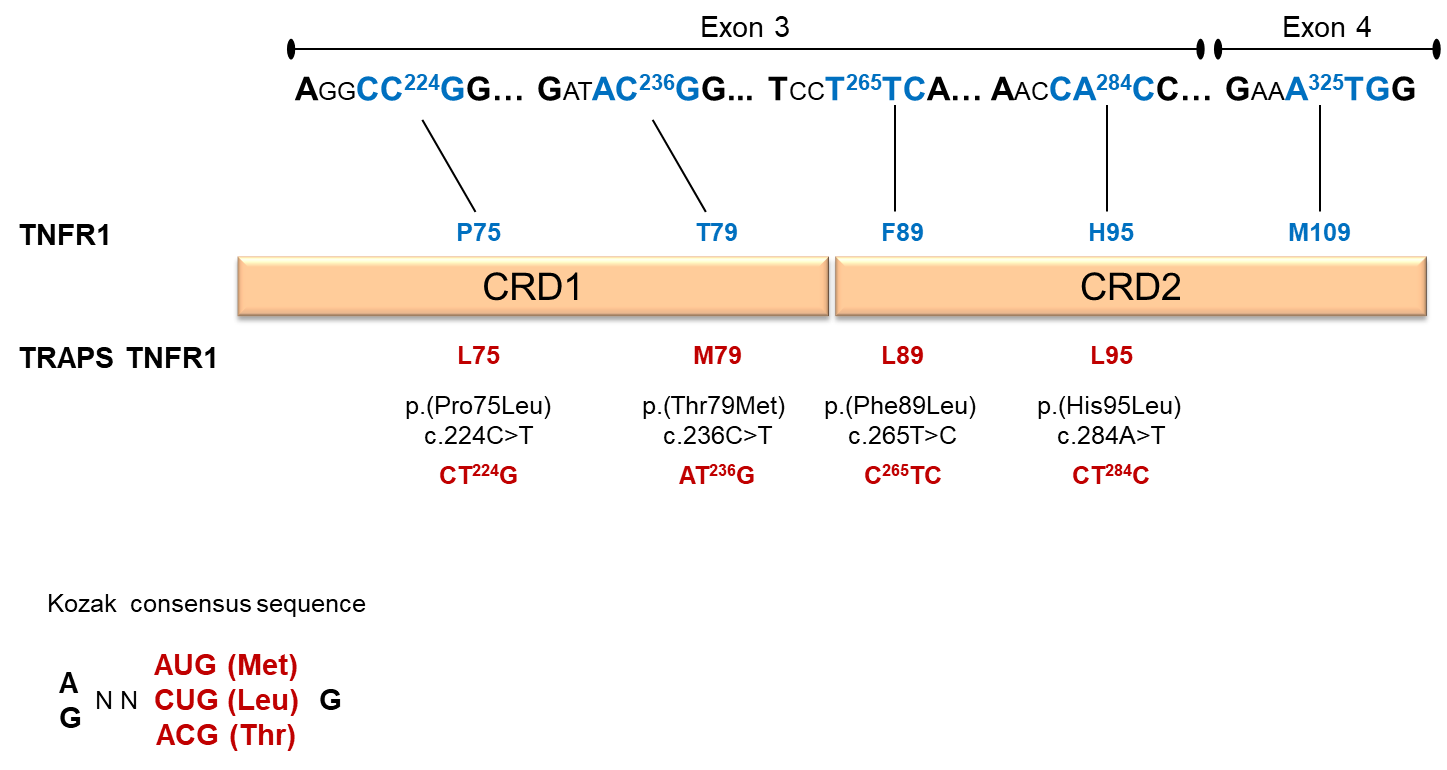
**

**Supplementary Fig. S7**

**
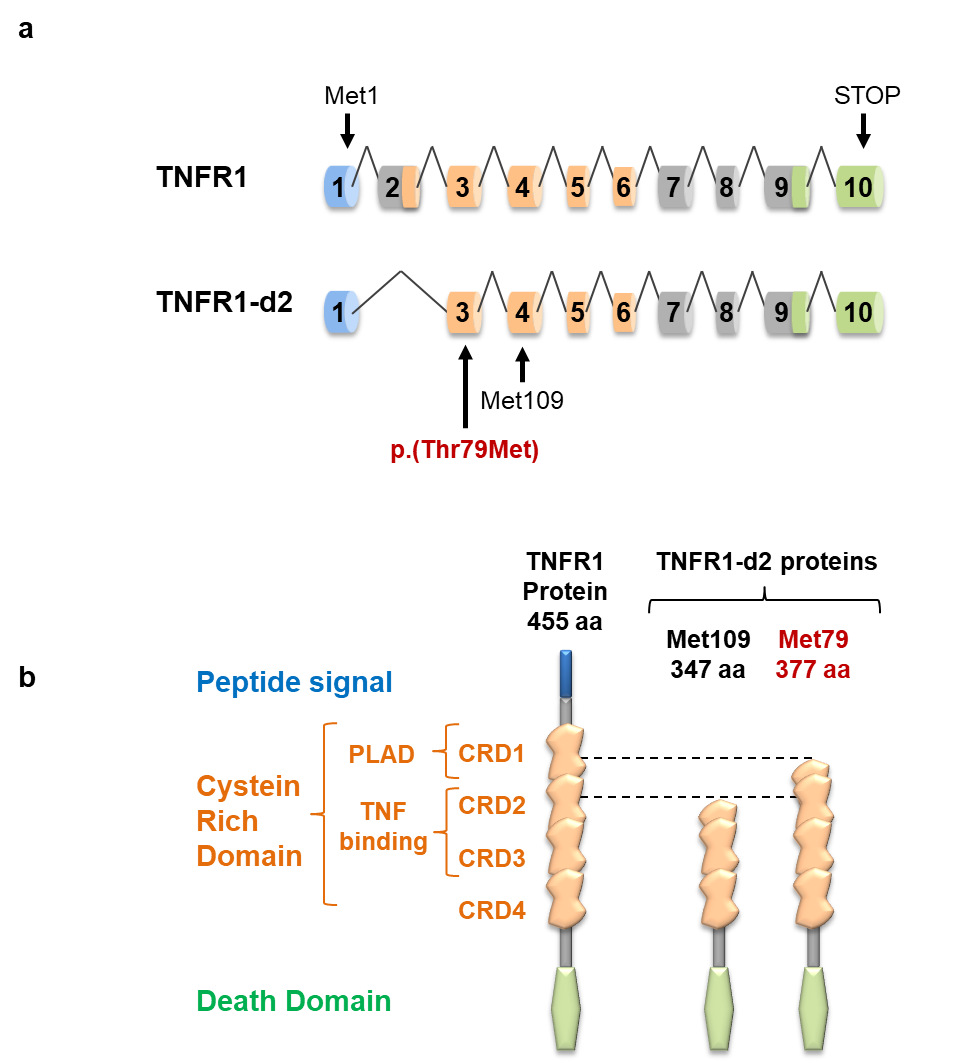
**

**Supplementary Fig. S8**


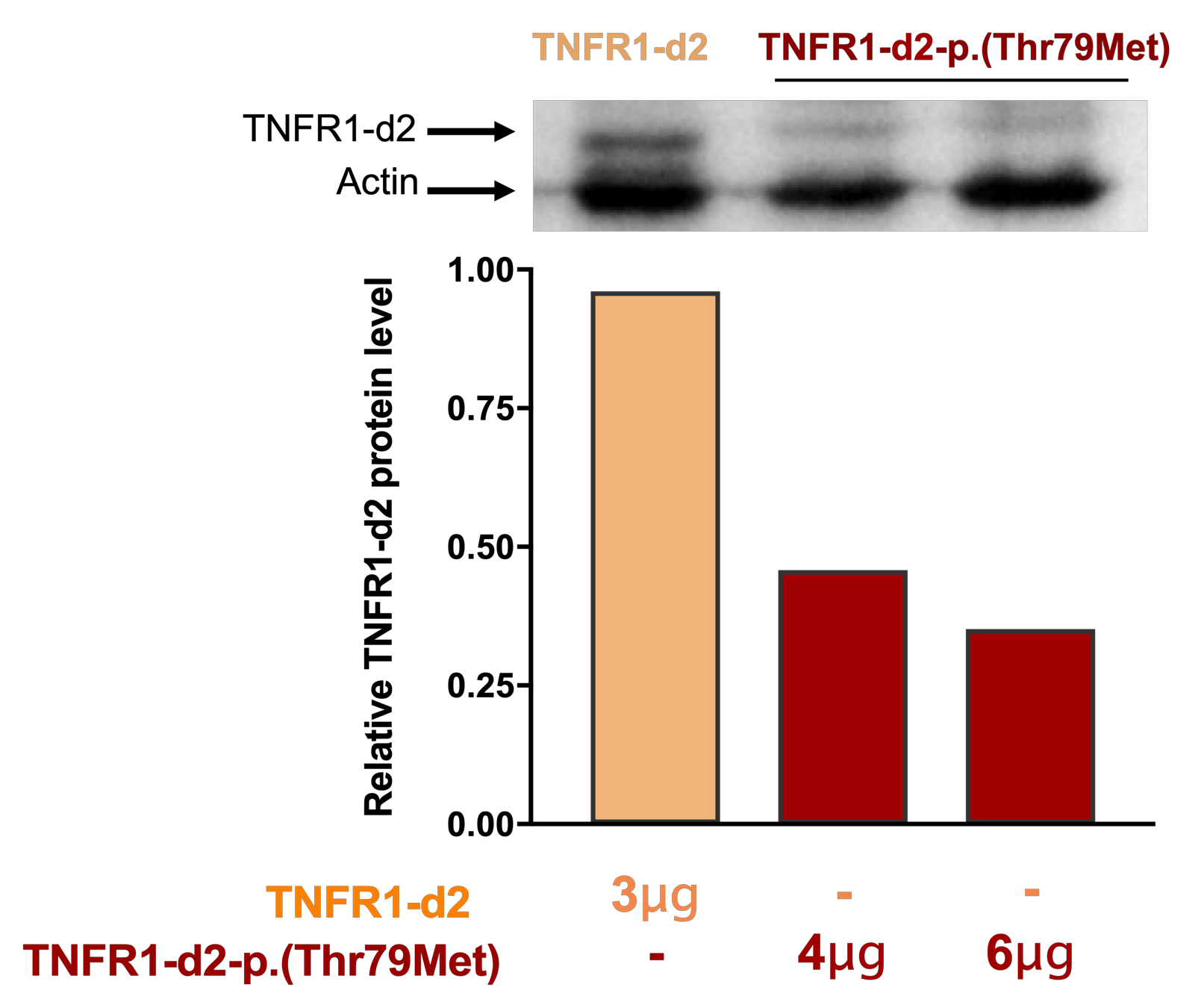


**a**

**b**

**References**

1. Rittore, C. *et al.* Identification of a new exon 2-skipped TNFR1 transcript: regulation by three functional polymorphisms of the TNFR-associated periodic syndrome (TRAPS) gene. *Ann. Rheum. Dis.* **73**, 290–297 (2014).

2. Dickie, L. J. *et al.* Involvement of X-box binding protein 1 and reactive oxygen species pathways in the pathogenesis of tumour necrosis factor receptor-associated periodic syndrome. *Ann. Rheum. Dis.* **71**, 2035–2043 (2012).

3. Martineau, Y. *et al.* Internal ribosome entry site structural motifs conserved among mammalian fibroblast growth factor 1 alternatively spliced mRNAs. *Mol. Cell. Biol.* **24**, 7622–7635 (2004).

4. Créancier, L., Morello, D., Mercier, P. & Prats, A. C. Fibroblast growth factor 2 internal ribosome entry site (IRES) activity ex vivo and in transgenic mice reveals a stringent tissue-specific regulation. *J. Cell Biol.* **150**, 275–281 (2000).

5. Huez, I. *et al.* Two independent internal ribosome entry sites are involved in translation initiation of vascular endothelial growth factor mRNA. *Mol. Cell. Biol.* **18**, 6178–6190 (1998).

6. Mathieu, M. *et al.* The glucocorticoid receptor gene as a candidate for gene therapy in asthma. *Gene Ther.* **6**, 245-252 (1999).
